# Traversing the Links between Heavy Metal Stress and Plant Signaling

**DOI:** 10.3389/fpls.2018.00012

**Published:** 2018-02-05

**Authors:** Siddhi K. Jalmi, Prakash K. Bhagat, Deepanjali Verma, Stanzin Noryang, Sumaira Tayyeba, Kirti Singh, Deepika Sharma, Alok K. Sinha

**Affiliations:** Plant Signaling, National Institute of Plant Genome Research, New Delhi, India

**Keywords:** calcium signaling, chelators, heavy metals, hormones, MAPKs, metal transporters, metallothioneins, microRNAs

## Abstract

Plants confront multifarious environmental stresses widely divided into abiotic and biotic stresses, of which heavy metal stress represents one of the most damaging abiotic stresses. Heavy metals cause toxicity by targeting crucial molecules and vital processes in the plant cell. One of the approaches by which heavy metals act in plants is by over production of reactive oxygen species (ROS) either directly or indirectly. Plants act against such overdose of metal in the environment by boosting the defense responses like metal chelation, sequestration into vacuole, regulation of metal intake by transporters, and intensification of antioxidative mechanisms. This response shown by plants is the result of intricate signaling networks functioning in the cell in order to transmit the extracellular stimuli into an intracellular response. The crucial signaling components involved are calcium signaling, hormone signaling, and mitogen activated protein kinase (MAPK) signaling that are discussed in this review. Apart from signaling components other regulators like microRNAs and transcription factors also have a major contribution in regulating heavy metal stress. This review demonstrates the key role of MAPKs in synchronously controlling the other signaling components and regulators in metal stress. Further, attempts have been made to focus on metal transporters and chelators that are regulated by MAPK signaling.

## Introduction

**Figure 1:**
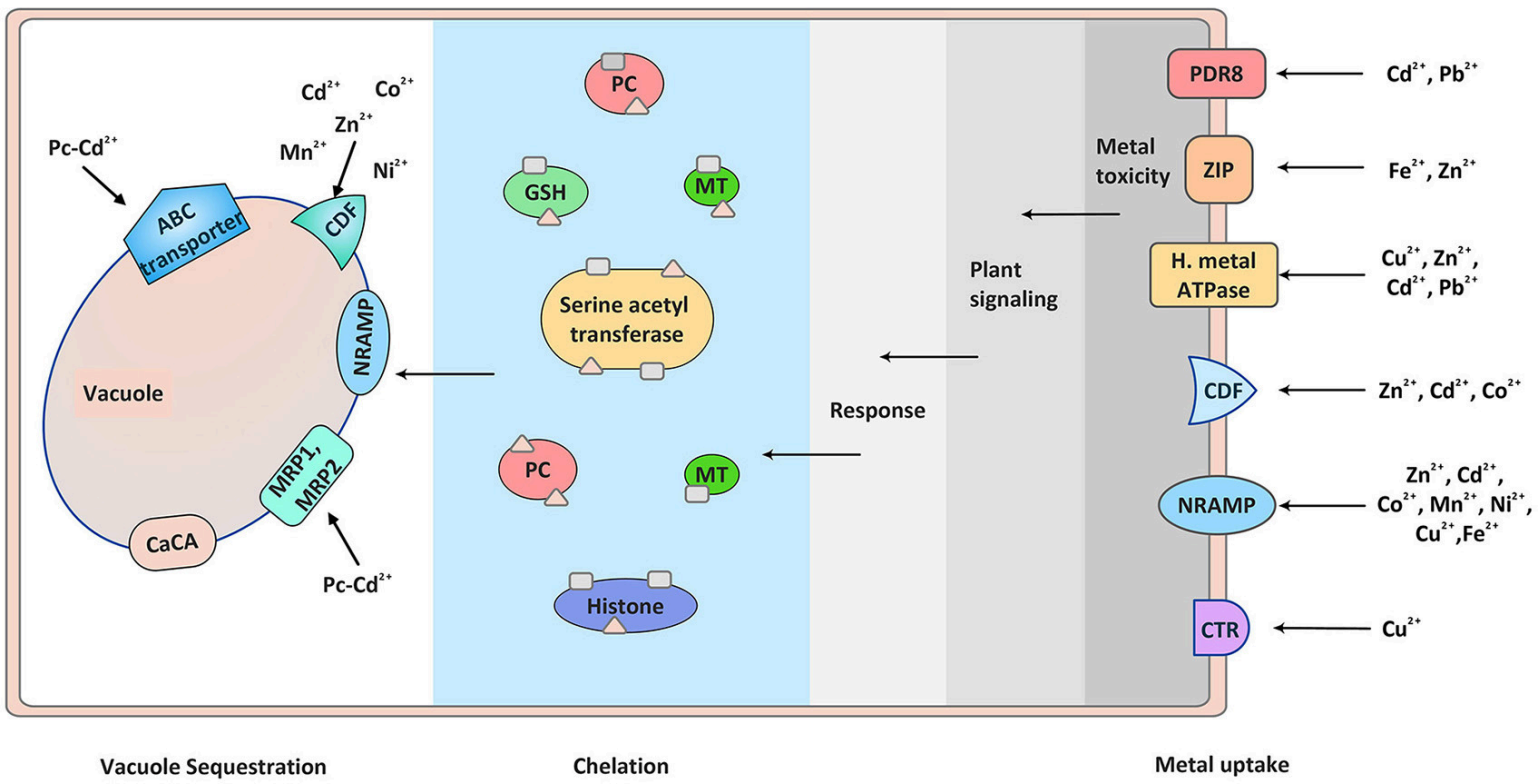
Metal detection, plant signaling, and sequestration. Different transporters are involved in metal ion uptake. Elevated level of heavy metals triggers different signaling modules which transmit the signals inside cell, thus triggering defense response. The toxicity of these metals inside the cell is sequestered by metal chelators like phytochellatins and metallothionines. The chelated metals are then ultimately transported to the vacuoles with the help of metal transporters present on the vacuole membrane. PC, phytochelatins; MT, metallothionines; GSH, Glutathiones.

**Figure 2:**
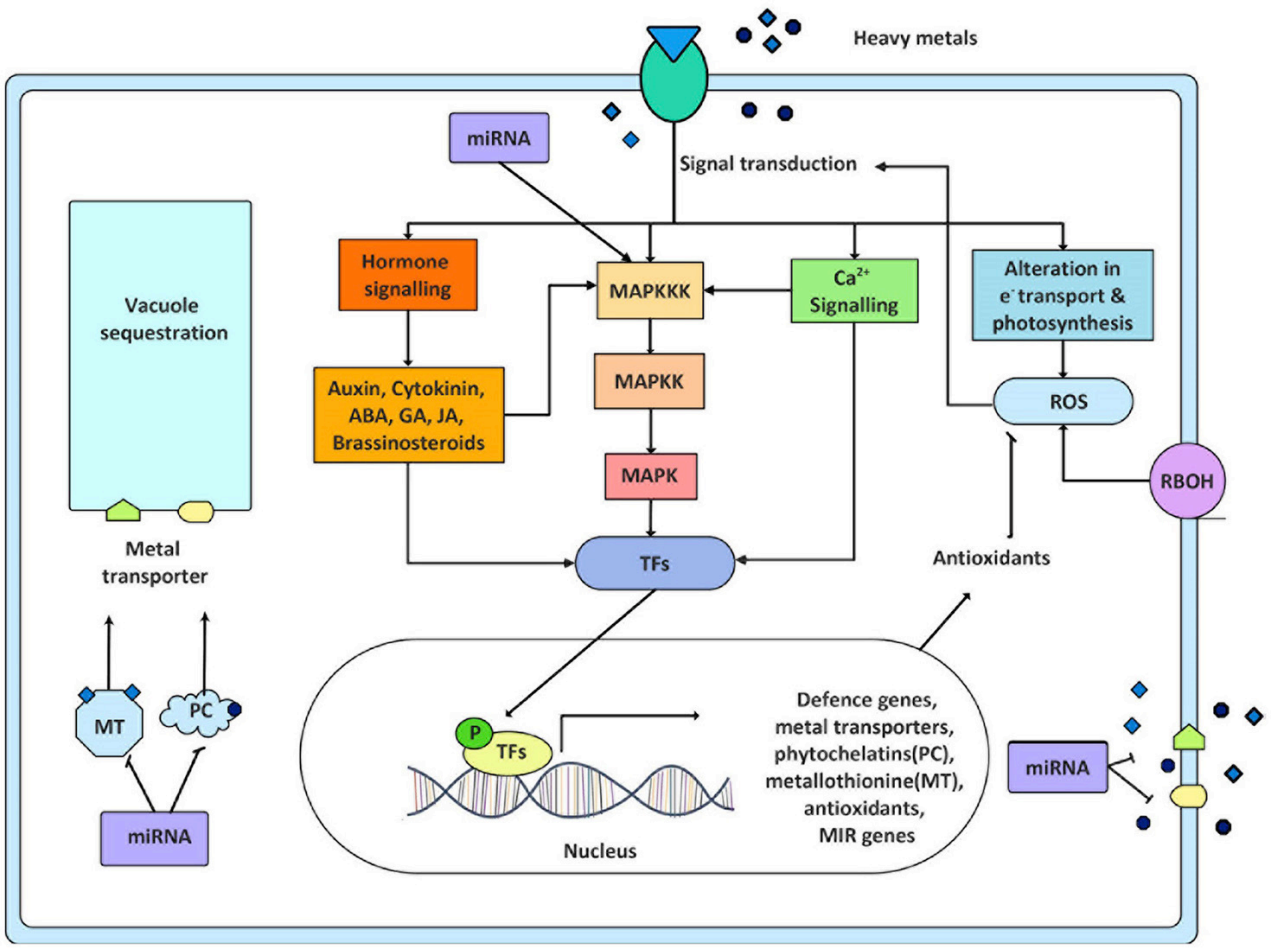
Crosstalk of signaling pathways and its ultimate response in heavy metal stress. This figure displays the involvement of several signaling components working during metal stress. Sensing of significant level of heavy metals by plants initiates signaling network causing activation of various metal responsive transcription factors. These transcription factors (TFs) regulate the expression of metal responsive and other stress related genes ultimately helping the plant to counteract stressed situation. These stress related genes are mainly metal transporters, phytochellatins, metallothionine, antioxidant genes, and miRNA genes (MIR genes). The ROS produced in response to metal stress either by respiratory burst oxidase homolog (RBOH) activity or by alteration in electron transport is also known to activate signal transduction. This figure also exhibits the crosstalk between different signaling modules and the feedback regulation of MAPK cascade by miRNA. P = phosphorylated.

Heavy metals are essential to life only in trace amount while their excess amount causes cellular damage. The heavy metals present in environment affecting the growth of many organisms are iron (Fe), arsenite (As^III^), arsenate (As^V^), cadmium (Cd), chromium (Cr), lead (Pb), copper (Cu), mercury (Hg), aluminum (Al), etc. These metals have not only known to perturb animal kingdom but also plant kingdom. Their damaging impact on our agriculture has also been very well-documented (Tchounwou et al., 2012). At cellular level elevated quantity of heavy metals imposes damage by wide number of mechanisms. The most common mechanism is the production of reactive oxygen species (ROS) inducing oxidative stress, while others are inactivation of biomolecules by displacement of essential metal ions or by blocking essential functional groups (Stohs and Bagchi, 1995). Metals like As, Cd, Cr, Pb, Hg are able to work by displacing essential metal ions or blocking functional groups. Metals like Fe and Cu, which are redox active, generate ROS directly through redox reactions; in contrast, other metals like Pb, Cd, Ni, Al, Mn, and Zn generate ROS by indirect mechanisms. The indirect mechanism of ROS production includes stimulation of ROS producing enzymes like NADPH oxidases or displacing essential cations from the binding sites of enzymes and inhibiting their activities. ROS at normal physiological level play essential role however its enhanced generation deteriorates functioning of cell (Cuypers et al., 2009). Plants show defense against these heavy metal ions by adsorbing them on to the chelating molecules [for e.g., phytochelatins (PCs), metallothionines, etc.] and by sequestration into the vacuoles (Figure 1). Many of the defense responses (not all) shown by the plants are due to the major contribution by signaling cascades, which perceive the signal from upstream receptors and transmit into the nucleus, thus regulating several defense related genes. The receptors that are known to perceive the signals and are well studied in plant stress and development include receptor like protein kinases (RLKs), flagellin sensitive 2 (FLS2), EF-Tu receptor (EFR), ethylene resistance1/2 (ETR1/2), salt intolerance 1 (SIT1), ERECTA (ER), etc. (Rodriguez et al., 2010; Sinha et al., 2011; Jalmi and Sinha, 2015). The major signaling networks working in metals stresses in addition to the other environmental stresses are calcium signaling, hormone signaling and MAPK signaling. Calcium signaling employs multitude of calcium sensing proteins like Calmodulins (CaMs), CaM like proteins (CMLs), Calcineurin B-like proteins (CBLs), and Ca^2+^-dependent protein kinases (CDPKs) that bind to Ca^2+^ and trigger different downstream signaling pathways (Luan et al., 2002; Sanders et al., 2002; Dodd et al., 2010; Steinhorst and Kudla, 2014). In case of hormone signaling there are different plant hormones that play role in metal stress response (Cao et al., 2009; Vitti et al., 2013; Chen et al., 2014; Zhao et al., 2014). Of the several signaling modules, the most predominant and complex is the mitogen activated protein kinase (MAPK) signaling composed of three-tier phosphorylation module MAPKKKs (Mitogen Activated Protein Kinase Kinase Kinase), MAPKKs (Mitogen Activated Protein Kinase Kinase), and MAPKs (Mitogen Activated Protein Kinase) (Hamel et al., 2006). MAPKs are substantially known in providing tolerance against biotic and abiotic stress (Rodriguez et al., 2010; Rao et al., 2011; Sinha et al., 2011) (Figure 2).

During environmental stress plants exhibit molecular response that helps them to adapt during various environmental calamities. Plant's molecular response to metal stress is signified by the synthesis of signaling molecules and stress-related proteins like metal transporters and chelators. They tackle the heavy metal toxicity by chelating and sequestering them in the plant vacuoles, serving as temporary storage of essential as well as toxic metabolites (Sharma and Dietz, 2006; Verbruggen et al., 2009; Mendoza-Cózatl et al., 2011) (Figure 1). Based on this ability plants are now being widely used for removing heavy metals contamination from the environment by process of phytoremediation (Salt et al., 1998).

Transport of heavy metals required for their relocation is performed by transporters localized in the parenchyma cells of xylem and companion cells of phloem. Majority of loading and unloading of the metal ions in xylem and phloem is done by the transporters. Prominent groups of transporters maintaining physiological concentration of heavy metals are: zinc–iron permease (ZIP), heavy metal transport ATPase (CPx- and P1B-ATPase), natural resistant associated macrophage protein (NRAMP), cation diffusion facilitator (CDF), and ATP-binding cassette (ABC) transporters, present at plasma membrane and on tonoplast membrane of cell (Park et al., 2012; Singh S. et al., 2015) (Figure 1). In addition, Cys-rich metal binding peptides like PCs or metallothionines, nicotinamide, and glutathione are also important players of metal transport (Figure 1). Studies suggesting the role of metal transporters and Cys-rich metal binding peptides in arsenic metal uptake, transport, and detoxification have been very well-described by Kumar et al. (2015). Apart from the transporters and chelators, vacuole sequestration capacity (VSC) is very much important in metal allocation. Interaction between membrane localized transporters and ion chelators adjust the VSC in response to changing environment (Peng and Gong, 2014). The regulation of the VSC will decide the toxicity of heavy metals to the plants. It is important to study the regulatory mechanisms of VSC and its ultimate impact on metal transport and sequestration. Additionally, study of metals signal perception and transmission by the plants in regulating the metal transport is also important.

This review will majorly emphasize on impact and mechanism of action of heavy metals, different signaling modules and other regulators triggered by heavy metal stress, the impact of plant signaling on downstream defense responses and the fragmentary work performed on regulation of metal transporters by MAPKs that still remains unexplored in plants.

### Plant signaling in response to heavy metals

The inability of plants to escape from environmental stresses such as metal pollution has driven the evolution of multiple mechanisms to efficiently sense, respond, and therefore adapt to such stresses. Sensing of heavy metals by plants generates a response such as modulation of molecular and biochemical mechanisms of cell. Certainly, this response is evoked by important signal transduction network operated in plant cell formed by several signal transduction units. The ultimate response of plant is shown by synthesizing metal transporter proteins and metal binding proteins helping the plant to counteract excessive metal stress (Maksymiec, 2007; Peng and Gong, 2014; Singh S. et al., 2015).

In many crops, the early sign of metal toxicity is known to be similar to other environmental stresses like osmotic or dehydration stress, oxidative stress in addition to defects in nutrient balance, photosynthesis, and development (Chen et al., 2001; Yadav, 2010; Rucinska-Sobkowiak, 2016). This similarity of the response reflects interconnection between intricate signaling networks. The interplay and convergence of these signaling pathways finally results in regulation of various transcription factors activating several stress responsive genes. The genes that normally get regulated in context of metal stress include the genes for metal chelators and transporters (Singh S. et al., 2015) (Figure 2). Several signal transduction units operate in response to heavy metal toxicity, with different signaling pathways acting in response to different species and concentrations of metals. Some of these signaling pathways are discussed in detail below.

### MAPK signaling in heavy metal stress

MAPKs are some of the most important and highly conserved signaling molecules that function in response to many diverse stresses and during many developmental pathways (Sinha et al., 2011). MAPK cascade consists of three tier components MAPKKKs, MAPKKs, and MAPKs mediating phosphorylation reactions from upstream receptor to downstream target (Hamel et al., 2006). MAPK signaling mediates the transmission of stress related signals thus regulating large number of cellular processes (Hamel et al., 2006; Rodriguez et al., 2010). Among abiotic stresses, heavy metal stress has conferred profound effect on MAPK signaling pathways. MAPKs are known to be activated by perception of specific metal ligand and also by ROS molecules produced in the metal stress (Jonak et al., 2004; Smeets et al., 2013; Jalmi and Sinha, 2015).

There are plenty of reports showing the activation of MAPKs in response to heavy metals like Cd, Cu, and As (Jonak et al., 2004; Yeh et al., 2007; Ding et al., 2011; Rao et al., 2011; Smeets et al., 2013), however studies in response to other metals such as Pb, Zn, Fe are very scant. Likewise, in depth investigation to decipher a complete MAPK signaling cascade in response to specific metal stress still remains elusive. In Arabidopsis, the best-characterized MAPKs are MPK3 and MPK6, which are activated by diverse stimuli are also known to induced by CdCl_2_ and CuSO_4_ (Asai et al., 2002; Pitzschke et al., 2009; Liu et al., 2010; Takahashi et al., 2011; Sethi et al., 2014). Similarly in rice, OsMSRMK2 (OsMPK3 homolog), OsMSRMK3 (OsMPK7 homolog), and OsWJUMK1 (OsMPK20-4 homolog) transcripts increased in response to Cu^2+^ and Cd^2+^ treatment in leaves and roots (Yeh et al., 2007; Rao et al., 2011). In Alfalfa, activation of four distinct MAPKs: SIMK, MMK2, MMK3, and SAMK was demonstrated in response to CuCl_2_ or CdCl_2_. SAMK and SIMK are the orthologs of rice OsMPK3 and OsMPK6, respectively. Higher concentrations of CuCl_2_ induced the activity of SIMK, MMK2, and MMK3, and to a lesser extent of SAMK while CdCl_2_ showed a similar but delayed MAPK activation (Jonak et al., 2004). Copper-mediated induction of SIMKK specifically activated SIMK and SAMK and not MMK2 and MMK3 manifesting specificity in the signaling cascades in response to different metals (Jonak et al., 2004; Opdenakker et al., 2012) (Table 1).

**Table 1:** Signaling components involved in metal stress.

**Heavy metal**	**Plant**	**Signaling components**		
		**MAPK**	**Calcium**	**Hormone**
Cd	Arabidopsis	MEKK1, MPK3, MPK6 (Jonak et al., 2004; Liu et al., 2010)	Unknown	**Auxin:** ^2^*PAT1*, ^2^*CYP79B2*, ^2^*CYP79B3*, ^2^*YUCCA*, ^2^*GH3*, ^2^*TIR1*, ^1, 2, 3^PINs, ^2^*ABCB*, ^2^*ARFs*, ^3^AXR3/IAA17 (^1^Hu et al., 2013; ^2^Wang et al., 2015; ^3^Yuan and Huang, 2016) **Cytokinin:** *IPT, CKX* (Vitti et al., 2013) **Ethylene:** ^4, 5^*ACS*, ^5^*ACC*, ^4^*ETR2*, ^4^*ERF1,5*, ^5^*GSH1*, ^5^*GSH2* (^4^Weber et al., 2006; ^5^Schellingen et al., 2015)
	Rice	MAPK2, MPK3, MPK6, MSRMK3, WJUMK (Agrawal et al., 2003; Yeh et al., 2007; Rao et al., 2011)	Unknown	**Auxin:** *MAPK3/6/7, YUCCA, PINs, ARF*, and *IAA* (Zhao et al., 2014)
	*M. sativa*	SAMK, SIMK, MMK2, MMK3 (Jonak et al., 2004)	Unknown	Unknown
	*Zea mays*	MPK3 (Wang et al., 2010)	Unknown	Unknown
	Radish	Unknown	Ca^2+^/CaM (Rivetta et al., 1997)	Unknown
	*B. natalensis, R. crispus*	Unknown	Unknown	**Cytokinin:** PI-55, INCYDE (Gemrotová et al., 2013)
	Glycin max	Unknown	Unknown	**Ethylene:** ACS, MAPK2, MAPKK2 Chmielowska-Bak et al., 2014
Cu	Arabidopsis	MPK3, MPK6 (Liu et al., 2010; Schellingen et al., 2015)	Unknown	**Auxin:** ^2^*PAT1*, ^2^*CYP79B2*, ^2^*CYP79B3*, ^2^*YUCCA*, ^2^*GH3*, ^2^*TIR1*, ^2^*PINs*, ^2^*ABCB*, ^2^*ARFs*, ^1, 2^*DR5* (^1^ Peto et al., 2011; ^2^Wang et al., 2015) **Ethylene:** ^4^COPT5, ^3^*ACS*, ^3^*ERF* (^3^Weber et al., 2006; ^4^Carrió-Seguí et al., 2015)
	Rice	MAPK2, MPK3, MPK6, MSRMK3, WJUMK (Agrawal et al., 2003; Yeh et al., 2007; Rao et al., 2011)	Unknown	Unknown
	*M. sativa*	SIMKK, SAMK, SIMK, MMK2, MMK3 (Jonak et al., 2004)	Unknown	Unknown
As	Arabidopsis	Unknown	Unknown	**Auxin:** AUX1, PIN1, PIN2 (Krishnamurthy and Rathinasabapathi, 2013) **Ethylene:** ERFs (Fu et al., 2014)
	Rice	MKK4, MPK3, MPK4 (Rao et al., 2011)	CaM, CaM kinase, CaM like protein (Chakrabarty et al., 2009; Huang et al., 2012)	Unknown
	*B. juncea*	46Kda MAPK (Gupta et al., 2009)	Unknown	Unknown
Al	Arabidopsis	Unknown	Unknown	**Auxin:** ^1, 2^PIN2, ^2^*AUX1* (^1^Shen et al., 2008; ^2^Sun et al., 2010)
	Wheat	Unknown	Myosin, Calpain, Phospholipase C, Phospholipase A_2_ (Jones and Kochian, 1997)	Unknown
	*T. aestivum*	48Kda MAPK, 42Kda Protein kinase (Osawa and Matsumoto, 2001)	Unknown	**Ethylene:** ^3^ALMT1, ^4^ACS, ^4^ACO (^3^Tian et al., 2014; ^4^Yu et al., 2016)
	*M. sativa*	Unknown	Unknown	**Auxin:** *AUX1, PIN2* (Wang S. et al., 2016)
Hg	Arabidopsis	Unknown	Unknown	**Auxin:** *PAT1, CYP79B2, CYP79B3, YUCCA, GH3, TIR1, PINs, ABCB, ARFs* (Wang et al., 2015)
	Rice	MSRMK2, MSRMK3, WJUMK (Agrawal et al., 2003)	Unknown	**Ethylene:** *OsACS2, OsACO1, OsACO2, OsACO5* and *OsACO6*, 5 *MAPKKK*, 1 *MAPKK* and 2 *MAPK* (Chen et al., 2014)
	*M. sativa*	Unknown	Unknown	**Ethylene:** *ACCS, ACCO, AP2, ERF1* (Montero-Palmero et al., 2014)
Pb	Arabidopsis	Unknown	CNGC1 (Sunkar et al., 2000)	**Auxin:** *PAT1, CYP79B2, CYP79B3, YUCCA, GH3, TIR1, PINs, ABCB, ARFs* (Wang et al., 2015) **Ethylene:** EIN2 (Cao et al., 2009)
	Rice	34Kda, 40Kda & 42Kda MAPK (Huang and Huang, 2008)	CDPK-like Kinase (Huang and Huang, 2008)	Unknown
	tobacco	Unknown	CBP4 (Arazi et al., 1999; Sunkar et al., 2000)	Unknown
	*R. sativus*	*MAPKKK7, MAPK6, MAPK18, MAPK20* (Wang et al., 2013)	Unknown	Unknown
Zn	Arabidopsis	Unknown	Unknown	**Auxin:** *PAT1, CYP79B2, CYP79B3, YUCCA, GH3, TIR1, PINs, ABCB, ARFs* (Wang et al., 2015)
	Rice	34Kda, 40Kda & 42Kda MAPK (Lin et al., 2005)	Unknown	**Auxin:** *MAPK3/6/7, YUCCA, PINs, ARF*, and *IAA* (Zhao et al., 2014)
	Wheat	Unknown	Myosin, Calpain, Phospholipase A_2_ (Jones and Kochian, 1997)	Unknown
Cr	*Zea mays*	MPK5 (Ding et al., 2009)	Unknown	Unknown
	Foxtail millet	Unknown	TPC1, MRC5, CaM (Fang H. et al., 2014)	Unknown
Mn	Arabidopsis	Unknown	ECA1 (Wu et al., 2002)	Unknown
Ni	Arabidopsis	Unknown	Unknown	**Auxin:** *PAT1, CYP79B2, CYP79B3, YUCCA, GH3, TIR1, PINs, ABCB, ARFs* (Wang et al., 2015)
	Tobacco	Unknown	CBP4 (Arazi et al., 1999)	Unknown
Ba	Faba bean	Unknown	Ca^2+^ channels (Hamilton et al., 2001)	Unknown
B	Barley	Unknown	Calmodulin, Ca^2+^- binding proteins (Tombuloglu et al., 2015)	Unknown

Beside the activation of MAPKs by Cu^2+^ and Cd metals, there are several other heavy metals that cause the activation of MAPKs, but are not studied in detailed aspect. In yeast, Al^3+^ tolerance was provided by over-expression of a MAP kinase gene in Al^3+^-sensitive mutant, indicating the association of MAPK with Al^3+^-resistance (Schott and Gardner, 1997). Similarly, in wheat root apex Al^3+^ treatment led to the activation of 48-kDa MAPK, playing significant role in transmitting Al related signals and Al-resistant in wheat (Mossor-Pietraszewska, 2001). In rice, a 42-kDa MAPK found to activate myelin basic protein (MBP) in response to iron. Pre-treatment of rice roots with an antioxidant, glutathione (GSH), decreased iron-induced root cell death, and MAPK activation, demonstrating the involvement of ROS induced MAPK activation in iron-triggered signaling (Tsai and Huang, 2006). Although, Zn is a non-redox metal, however MAPK activation by Zn results from the activation of oxidative stress in rice. Zn stimulates a rapid activation of MBP by three MAPKs with approximate molecular weights of 34, 40, and 42 kDa in rice roots (Lin et al., 2005). Pb stress leads to the upregulation of four MAPKs such as MAPKKK7, MAPK6, MAPK18, and MAPK20 in radish (Wang et al., 2013). Arsenite severely affect the growth of rice seedlings. OsMKK4 and OsMPK3 transcripts were found to be induced in arsenite treated rice leaves and roots (Table 1). *In-silico* homology modeling and docking analysis supported OsMKK4–OsMPK3 interaction (Rao et al., 2011), suggesting the role of this MAPK module in arsenic stress. Accumulating evidences suggest that metal ions such as arsenic and chromium are able to induce reactive oxygen and nitrogen species, thereby altering nitric oxide (NO) induced cell signaling. NO has been shown to modulate the activity of MAPK, NO donors, and recombinant NOS were shown to cause the activation of SIPK (Rao et al., 2011).

Heavy metal induced ROS production is already known in plants and the role of these ROS molecules in activating MAPK signaling is very well-accepted. In Arabidopsis, two important completely characterized MAPK cascade MEKK1-MKK4/5-MPK3/6 (Asai et al., 2002) and MEKK1-MKK2-MPK4/6 are known to work downstream of ROS, participating in both abiotic and biotic stress signaling (Pitzschke et al., 2009; Jalmi and Sinha, 2015). Apart from this, MAPK cascades also exert positive feedback regulation on ROS production. A cascade OXI1-MPK6 activated by ROS also positively regulates ROS production (Asai et al., 2008). MEKK1-MKK4-MPK3/6 is known to act upstream of NADPH oxidase stimulating ROS production in pathogen attack and H_2_O_2_ produced is in turn known to activate MPK3 and MPK6 (Kovtun et al., 2000). These studies provide a speculation and link of MAPK cascades that might work in different metal stress depending upon the activation by the ROS molecules produced. Furthermore, MEKK1-MKK4/MKK5-MPK3/MPK6 module working downstream of receptor FLS2 and receptor like kinase (RLKs), eventually giving resistance against pathogen is very well known (Asai et al., 2002). These RLKs are also reported to be regulated by Cd^2+^ speculating the involvement of similar MAPK cascade working downstream of RLK in metal stress. Recent study reported that MEKK1-MKK5-MPK6 mediates salt induced expression of iron superoxide dismutase gene further inducing ROS production. Iron superoxide dismutases (Fe-SOD) are the metal binding SOD and require Fe^3+^ metal ion as cofactor (Myouga et al., 2008; Xing et al., 2015). These reports suggest involvement of MAPKs in mediating metal stress however a detail study of a complete MAPK cascade working in heavy metal stress is required.

### Calcium signaling in heavy metal stress

The calcium ion (Ca^2+^) as corroborated by different studies acts as a universal secondary messenger in the normal functioning of plants as well as in response to various environmental stresses (Sanders et al., 2002). The cytosolic free Ca^2+^ concentration changes in response to various stress stimuli triggering complex interactions and signal transduction pathways (Rudd and Franklin-Tong, 2001). This transient increase in the cytosolic concentration is perceived by highly sensitive calcium sensing proteins that mediate this chemical signal into a biological response. Plants harbor myriads of calcium sensing proteins such as Calmodulins (CaMs), CaM like proteins (CMLs), Calcineurin B-like proteins (CBLs), and Ca^2+^-dependent protein kinases (CDPKs) that bind to Ca^2+^ and trigger different downstream signaling pathways (Luan et al., 2002; Sanders et al., 2002; Steinhorst and Jörg, 2003; Dodd et al., 2010).

Multiple studies in different plant species, such as chickpea, *Glycine max, Vitis vinifera*, and tomato have been carried out to discern the contribution of Ca^2+^-binding like proteins and Ca^2+^ sensing proteins in augmented tolerance to various abiotic stresses (Tripathi et al., 2009; Li Z. Y. et al., 2012; de la Torre et al., 2013). There have been reports demonstrating that the application of Ca^2+^ exogenously can modulate the physiological and biochemical responses in order to alleviate the heavy metal stress. The activity of antioxidant enzymes such as ascorbate peroxidase, glutathione reductase, and superoxide dismutase has been shown to be enhanced upon the application of exogenous Ca^2+^ (Ahmad et al., 2015). Though there have been several reports that substantiate the role of Ca^2+^ and Ca^2+^-dependent signaling pathways in imparting tolerance to heavy metal stresses in plants, our understanding of the mechanisms by which these responses are regulated is still meager and invites further elaboration.

Cd is one of the heavy metals physiochemical properties very similar to that of calcium (Choong et al., 2014). This naturally results in an exchangeability of the two ions in Ca^2+^ binding proteins and studies have provided evidence that Cd displaced Ca^2+^ from its binding sites in calmodulin, sarcolemma and troponin C *in vitro* (Langer and Nudd, 1983; Chao et al., 1984; Ellis et al., 1984). The high similarity in the ionic radii of Ca^2+^ and Cd indicates a possibility of Cd uptake through receptor- or voltage-gated Ca^2+^ channels and this uptake could possibly be inhibited (at least to some extent) by blocking the Ca^2+^ channels (Choong et al., 2014). Plants exposed to cadmium exhibit a higher level of intracellular Ca^2+^, inducing adaptive mechanisms in order to mitigate the toxic effects of the heavy metal (Yang and Poovaiah, 2003). One of the mechanisms used for the increase in Ca^2+^ level is the production of IP_3_ which triggers the release of sequestered calcium from the intracellular calcium reserves as indicated in few reports (Smith et al., 1989). A study of *Brassica juncea* in consistence with the above report proves that application of Ca^2+^ attenuates the toxicity and makes the plant withstand the deleterious effects of Cd consequently improving the growth and seed quality of the plants (Ahmad et al., 2015). Moreover, studies on different plant species have demonstrated that exogenous application of calcium and silica, calcium and spermidine and Ca^2+^ and/or K^2+^ can promote the alleviation of cadmium toxicity and reduction in metal accumulation (Siddiqui et al., 2012; Srivastava et al., 2015; Gong et al., 2016).

An interesting study on *Arabidopsis* seedlings has shown that Ca^2+^ mitigates the toxic effects of Cd through maintaining auxin homeostasis indicating a crosstalk between signaling pathways in order to combat heavy metal stress (Zhao et al., 2015). Moreover, studies on yeast cells have proposed the role of Ca^2+^-ATPases (Pmr1p and Pmc1p) of vacuolar and Golgi membrane in coping with Cd toxicity. This is achieved through cooperation with a Glutathione-conjugated transporter Ycf1p whose activity is controlled by phosphorylation once again insinuating an interface between different signaling pathways in response to environmental stresses (Mielniczki-Pereira et al., 2011).

The Ca^2+^/Calmodulin system is also involved in response to toxicity mediated by heavy metals other than Cd such as Pb^2+^ and nickel (Ni^2+^) (Ahmad et al., 2015). It was demonstrated by Arazi et al. that transgenic tobacco plants expressing the plasma membrane associated NtCBP4 (*Nicotiana tobacum* calmodulin-binding protein) exhibit higher levels of tolerance to Ni toxicity. Contrastingly, the same plants were found to be hypersensitive to Pb^2+^, depicting an exclusion of Ni^2+^ and augmented accumulation of Pb^2+^ as compared to wild type plants (Arazi et al., 1999) (Table 1).

In foxtail millet (*Setaria italica*), hydrogen sulfide was found to interact with Ca^2+^ signaling in imparting improved tolerance to Chromium (Cr VI^IV^)-mediated heavy metal stress. It has also been discerned that Ca^2+^ provides tolerance against Cr stress by enhancing the activity of antioxidant enzymes (Fang H. et al., 2014). Additionally, an involvement of CDPKs has also been suggested through transcriptional profiling of the rice roots exposed to long or short durations of Cr stress. Increasing Cr(VI) concentration was found to be correlated with an increase in CDPK-like protein activity, reflecting the role of Ca^2+^ signaling in the stress response (Huang et al., 2014) (Table 1).

Differential expression of Calmodulins in response to arsenic stress indicates the possible role of Ca^2+^ signaling components in the stress response (Chakrabarty et al., 2009). Besides, a study has demonstrated that cytosolic free Ca^2+^ played a key role in the regulation of root activity, metal contents and biomass in close relation to lanthanum (La) dose and acid rain strength. The adverse effects on the roots caused by acid rain could be alleviated by low concentrations of La^III^ and synergistic effects on the roots were observed upon combined exposures at higher concentrations of La(III) and acid rain (Zhang et al., 2016). The release of intracellular Ca^2+^, the subsequent activation of Ca^2+^ channels and the generation of H_2_O_2_ was observed in response to elevated levels of Cu^2+^ in the marine alga, *Ulva compressa*. It was evidenced that the gene expression of antioxidant system is regulated via cross-talk among the various cellular signals and levels of Ca^2+^, NO, and H_2_O_2_ (González et al., 2012).

It is established that in response to environmental changes CDPK work together with MAPK for transmission of signals to adapt against changing environment (Takahashi et al., 2011; Wurzinger et al., 2011; Opdenakker et al., 2012) (Figure 2). A CDPK, CPK18 was found to be an upstream kinase of MPK5 in rice, wherein MPK5 was phosphorylated on Threonine 14 and 32 by CPK18 (Xie et al., 2014). Also, MKK3 together with Ca/CaM is known to activate MPK8, which negatively regulates the expression of RBOHD (NADPH oxidase) in response to mechanical stress (Takahashi et al., 2011). A study suggest that Ca^2+^ is involved in Pb^2+^-mediated cell death and triggering of MAPK activity via CDPK pathway by enhancing the activity of CDPK like kinase (Huang and Huang, 2008). Besides this, the role of calmodulins has been reported to modulate MAPK signaling pathway (Tebar et al., 2002), which defines a possibility of their interplay in response to metal stress. All these findings outline a vital function for the Ca^2+^ regulatory loop, which is critical for maintaining the redox homeostasis of the cell and ion balance in response to heavy metal stress. In animals this crosstalk has been elaborately studied in metal stress than in plants. Hence, it will be highly advantageous to study the importance of this signaling crosstalk and further the regulation of Ca^2+^ signaling in heavy metal stress in plants.

### Hormone signaling in heavy metal stress

The root architecture is of great importance in plant grown in metal-polluted areas, as the remodeling of root architecture in response to metals can be used as a strategy to escape from heavy metal stress. Interestingly, auxin, ethylene, and cytokinin modulate patterning (Vanstraelen and Benková, 2012) and lateral root formation (De Smet et al., 2015). Thus, there are several studies reporting the involvement of these phytohormones in remodeling of the root system architecture in response to heavy metal stress.

Auxin is an essential plant growth hormone playing role in developmental as well as environmental stress responses. It directly affects plant responses to metal stresses by modulating auxin homeostasis including auxin stability, transport, and redistribution (Potters et al., 2007). Basipetal auxin transport through the outer root cell layers is mediated by AUX1 and PIN2 (Marchant et al., 1999; Rashotte et al., 2000). The regulation of auxin signaling in heavy metal stress is evident by various studies conducted over the years. Recently, it was reported that in response to metal stress plants regulate the location and accumulation of auxin by differential and dynamic expression of auxin-related genes like Phosphoribosyl Anthranilate Transferase 1 (PAT1), CYP79B2 and CYP79B3, YUCCA (YUC), Gretchen Hagen (GH3) genes, (TIR1), PIN family, and ABCB family (Wang et al., 2015) (Table 1). Cu^2+^ toxicity in Arabidopsis leads to changes in auxin and cytokinin accumulations and mitotic activity within the primary and secondary root tips (Lequeux et al., 2010). It is also reported that in excess of Cu^2+^ lack of auxin leads to an increase in NO levels thereby diminishing root elongation (Peto et al., 2011). This inhibition of primary root elongation by Cu is also due to the modulation of auxin redistribution by PIN1 (Yuan and Huang, 2016). Further, Al was also studied to inhibit root growth by inhibiting the transport of PIN2 vesicles from plasma membranes to endosomes, further disturbing IAA synthesis in apical buds and imbalance of IAA transportation and distribution in roots (Shen et al., 2008; Wang S. et al., 2016). It was further revealed that *aux1-7* and *pin2* mutants exhibited better tolerance to Al^3+^ than wild-type plants implying the plausible involvement of AUX1 and PIN2 proteins in Al^3+^ induced inhibition of root elongation. Apart from this, Cd disrupts the maintenance of auxin homeostasis in Arabidopsis seedlings by increasing IAA oxidase activity and altering the expression of several auxin biosynthetic and catabolic genes (Hu et al., 2013). Cd-mediated up-regulation of biosynthesic gene *NITRILASE* (*NIT*) resulted in increased IAA concentration in Arabidopsis roots promoting lateral root growth, thus protecting roots from Cd (Vitti et al., 2013). Moreover, recent report revealed the inhibition of root meristem growth through Cd-induced NO accumulation, which in turn represses auxin transport and stabilizes AUX/IAA proteins to repress auxin signaling (Yuan and Huang, 2016). A positive role for auxin transport through AUX1 on plant tolerance to As stress via ROS-mediated signaling was also disclosed in a study (Krishnamurthy and Rathinasabapathi, 2013) (Table 1).

MAPK signaling is established in influencing auxin signaling and its transport (Mockaitis and Howell, 2000). A captivating report unveiled the interplay of auxin/cytokinin and MAPKs, in which OsMKK4/5-OsMPK3/6 was elucidated as key player in auxin/cytokinin interaction regulating the expression pattern of OsPIN1b/9 (Singh P. et al., 2015). However, the involvement of MAPK signaling regulating auxin response in metal stress is still uncertain. Appealing study performed in rice displayed relationship between auxin signaling and MAPK signaling in Cd stress. It was analyzed that expression of most of the key genes of auxin signaling including YUCCA, PIN, ARF, IAA, and cell cycle related genes was negatively regulated by MAPK in Cd stress (Zhao et al., 2014). This certainly implicates the major role of MAPK signaling in regulating auxin signaling in heavy metal stress.

Cytokinins (CKs) are N6-prenylated adenine derivatives involved in the regulation of plant growth and development and in biotic and abiotic stresses (Perilli et al., 2010). There are reports of CKs in plants activated upon heavy metal stress that are able to alleviate heavy metal induced toxicity. The inhibition of photosynthetic pigment and chloroplast membranes by Cd was restored by CKs, increasing photosynthetic capacity and primary metabolite levels (Piotrowska-Niczyporuk et al., 2012). Exogenous kinetin application can also modulate antioxidant enzyme activity, proline, free amino acids, and soluble sugars that counteracted Cd caused inhibitory effects on growth and photosynthesis (Al-Hakimi, 2007).

Ethylene (ET) is a gaseous plant hormone regulating various important growth aspects. It is biosynthesized by ACC synthase (ACCS) that convert AdoMet to ACC, while ACC oxidase (ACCO) catalyzes the conversion of ACC to ethylene. ACCS and ACCO are encoded by multigene families and regulated by many biotic and abiotic factors (Kende, 1993). Several reports support the role for ethylene in the regulation of plant metal stress responses. The effects of metal stress on ethylene production in plants are both metal and concentration dependent (Thao et al., 2015; Keunen et al., 2016) (Table 1). Major five ET synthesis genes from rice OsACS2, OsACO1, OsACO2, OsACO5, and OsACO6 (Chen et al., 2014) along with transcription factors AP2 and ERF1 from *Medicago sativa* (Montero-Palmero et al., 2014) were found to be upregulated in Hg treatment. However in rice, genes involved in cytokinin signaling (OsRR1, 3, 4, 6, and 11) were down regulated, suggesting both ET and CK may regulate the Hg-induced inhibition of rice root growth (Chen et al., 2014). Additionally, Cu and Al were also found to increase ACS transcript level in Arabidopsis, *Medicago truncatula* and *Lotus japonicus* (Weber et al., 2006; Sun et al., 2010). There was inhibition in root growth under Al stress which was correlated to enhanced ethylene production upon Al treatment (Sun et al., 2010). Recently, it was revealed that in wheat ET negatively regulates Al-induced efflux of malate ions using ET8, which is an important mechanism for Al tolerance (Tian et al., 2014; Yu et al., 2016) (Table 1).

Besides the effect of heavy metals on ethylene synthesis, they even exert effect on ethylene signaling. Cu treatment increases expression of number of ethylene responsive factors like *ERF1, ERF2*, and *ERF5* (Weber et al., 2006). Apart from these, Cd was exhibited to establish its role in regulating ethylene synthetic genes (ACS2 and ACS6) along with MAPK cascades, NO generation, and polyamine metabolism (Chmielowska-Bak et al., 2014; Schellingen et al., 2015) (Table 1).

Like the interplay of MAPK with auxin signaling, there are also evidences of involvement of MAPKs in ethylene biosynthesis and signaling, however its importance in metal stress is still unknown (Opdenakker et al., 2012). Two important MAPKs, MPK3 and MPK6 are known to be responsible for phosphorylation of ACS2 and ACS6, thus increasing ethylene production (Liu and Zhang, 2004; Li G. et al., 2012). This ACS2/6 is very well reported to be induced by metal stress. Further, putresciene, an essential signaling molecule involved in modulating plant resistance to Al stress by inhibiting ACS and ET production (Yu et al., 2016), was found to be regulated by AtMPK3/6 (Kim et al., 2013). Moreover, a MAPK cascade MKK9-MPK3/6 acting downstream of ethylene receptor CTR1 was found to control a key transcription factor EIN3 involved in ethylene biosynthesis (Xu et al., 2008; Yoo et al., 2008). Roots of rice plants exposed to Cr showed an increased expression of the *EIN3* and *EIN4* genes (Trinh et al., 2014) suggesting the putative role of this MAPK cascade heavy metal induced ET biosynthesis. Recently, Chmielowska-Bak et al. (2014) revealed that cadmium causes induction of ethylene responsive genes and MAPKs in soybean seedlings also suggesting an elevation in MAPKK2 gene expression. Further, Schellingen et al. (2015) suggested a link between MPK3/6 mediated ROS production and ethylene signaling during Cd stress in Arabidopsis.

### Regulation of micrornas during heavy metal stress

Besides the contribution of signaling pathways in transmitting heavy metal related stimuli and regulating the plant response, other regulators like small RNAs are majorly found to have profound effect on metal stress response. Small RNAs such as microRNAs are a 20–24 nucleotide non-coding RNAs that regulate the gene expression at post-transcriptional level by targeting mRNA degradation or by translation repression (Raghuram et al., 2014). It has been shown that different miRNA families are differentially regulated temporally as well as spatially, differing in concentration from species to species (He et al., 2016). All these data indicate that differential regulation of any miRNA depends upon the function of miRNA target, physiology, and metabolism of the plant.

**Figure 3:**
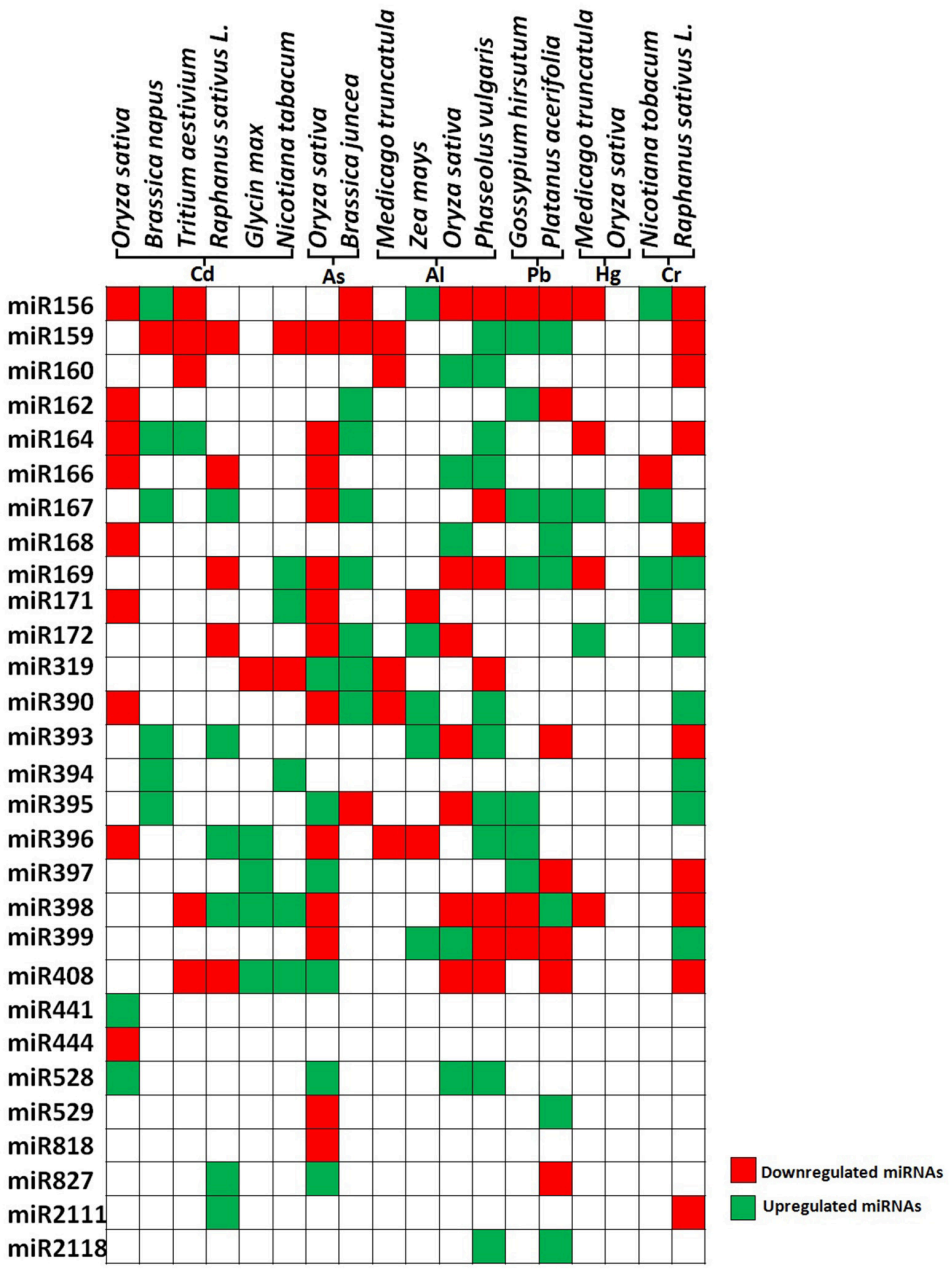
Differential expression of heavy metal responsive microRNAs in plants. The figure represents the data taken from genome wide study of differentially expressing miRNAs in different plant species. Green color and red color indicates up regulated and down regulated miRNAs respectively.

Recently genome wide, transcriptome analysis, and high throughput sequencing have been used to identify the microRNAs, which are responsive to heavy metal stress in many plant species. It has been shown that various conserved miRNAs are differentially regulated during the normal and stress conditions (Figure 3). Differential expression of miRNAs in heavy metal stress indicates the possible involvement of miRNAs in heavy metal stress detoxification and tolerance (Ding et al., 2011; Liu and Zhang, 2012; Zhou et al., 2012; Bukhari et al., 2015; Noman and Aqeel, 2017). Here, we have focused on studies showing differential expression of conserved miRNA in metal stress and regulation of signaling pathway by miRNAs or vice versa in response to heavy metals.

Plant exposed to excess concentration of Cd, employed differential regulation of miRNAs. For example in rice, Cd exposure modulates expression of various novel and evolutionarily conserved miRNAs. Upon exposure to Cd, miR441 expression was significantly upregulated while 12 miRNAs were found to be down-regulated. Among the down-regulated miRNAs, miR192 predicted to target ABC transporter, which is shown to be involved in heavy metal transport. Overexpression of miR192 significantly reduced rice seed germination and seedling growth under Cd stress compared to wild-type plants. This suggests that decrease in the amount of miR192 leads to the accumulation of ABC transcripts which eventually leads to Cd sequestration by ABC transporter during Cd stress (Ding et al., 2011; Tang et al., 2014; He et al., 2016) (Figure 3).

Contemplating these studies carefully we found that cadmium stress downregulates major miRNAs like miR159 and miR166 in most of the plant species (Figure 3). The targets of several Cd responsive miRNAs, including miR398 and miR408 have been shown to target the heavy metal detoxification genes. The Cu–Zn superoxide dismutase (CSD), is an essential enzyme for the detoxification of superoxide radicals and reduced accumulation of respective miRNAs lead to the accumulation of these scavengers during stress, hence protecting the plants against heavy metal induced oxidative damage. Recent studies have identified many As responsive miRNAs by deep sequencing in rice and mustard (Liu and Zhang, 2012; Srivastava et al., 2013; Pandey et al., 2015; Sharma et al., 2015) (Figure 3). They have reported that the expression of miR172 was significantly down-regulated whereas miR393, miR397, and miR408 were upregulated. Studies revealed that miR408 has a direct role in targeting Cu containing proteins or superoxide dismutase (Ma et al., 2015). Also, it has been reported that during heavy metal stress, ROS leads to the induction of lipid peroxidation and downregulation of miR397, which has been shown to target laccase. This may lead to positive regulation of lignin biosynthesis through the accumulation of laccase enzymes (Jones-Rhoades and Bartel, 2004) (Figure 3).

Aluminum (Al) is being considered as a major limiting factor for plant development interfering with cellular redox equilibrium. Similar to other metals, Al stress also downregulates most of the miRNAs in rice such as miR156, miR395, miR398, miR159 except miR399, miR166, miR168 which showed upregulation (Lima et al., 2011). Contrastingly, in maize the similar miRNAs showed upregulation except miR171 and miR396 which showed downregulation (Kong et al., 2014) (Figure 3). MicroRNAs also play a key role in metal complexation wherein two important genes ATP sulfurylase (APS) and SULTR2:1 were identified as targets of miR395 which is reported to induce by Al stress, Cd stress, and sulfur deficiency. Both of these genes lead to the production of GSH and PCs which are chief molecules in metal chelation (Matthewman et al., 2012) (Table 2).

**Table 2:** Heavy metal responsive conserved miRNAs, their targets, and functions.

**miRNAs**	**Targets**	**Target functions**	**References**
miR156	SQUAMOSA-PROMOTER BINDING PROTEIN (SBP)-like proteins (SPL)	Floral development	Schwab et al., 2005
miR159	MYB transcription factors	Floral development	Achard et al., 2004
miR160	ARF transcription factors	Auxin signaling, Floral organ development	Wang et al., 2005
miR162	DCL1	Micro RNA biogenesis	Xie et al., 2003
miR164	NAC, CUC genes	Drought resistance, Leaf margin serration	Nikovics et al., 2006; Fang Y. et al., 2014
miR165/166	HD-ZIP transcription factors, KANADI	Root development	Singh et al., 2017
miR167	ARF transcription factors	Auxin signaling	Wang et al., 2015
miR168	AGO1	MicroRNA pathway	Vaucheret et al., 2004
miR169	Nuclear factor Y	Drought resistance	Li et al., 2008
miR171	GRAS domain transcription factors/SCARECROW-like (SCL)	Floral development	Ma et al., 2014
miR172	APETELA2-like transcription factors	Transcriptional regulation, Developmental phase transition	Aukerman and Sakai, 2003
miR319	TCP family transcription factor	JA biosynthesis, Senescence	Schommer et al., 2008
miR390	Stress-responsive leucine-rich repeat receptor-like kinase(SRK), ARF	Cd stress tolerance	Fahlgren et al., 2006; Ding et al., 2016
miR393	TIR1, AFB family	Auxin signaling	Chen et al., 2011
miR394	LEAF CURLING RESPONSIVENESS (LCR)	Abiotic stress tolerance	Song et al., 2016
miR395	ATP sulfurylases (APS), ARABIDOPSIS SULFATE TRANSPORTER 68 (AST68)	Sulfate assimilation	Matthewman et al., 2012
miR396	GROWTH-REGULATING FACTOR(GRF) TFs, bHLH74	Cell proliferation regulation	Debernardi et al., 2012
miR397	Laccase	Lignin biosynthesis	Jones-Rhoades and Bartel, 2004
miR398	Cu–Zn superoxide dismutase (CSD)	ROS response	Jones-Rhoades and Bartel, 2004
miR399	ubiquitin-conjugating enzyme E2 24 (UBC24)/PHOS2	Phosphate starvation	Chiou et al., 2006
miR408	Cu containing proteins, Cu/Zn superoxide dismutase, Cu chaperon	Abiotic stress tolerance	Ma et al., 2015
miR441	Unknown	–	–
miR444	MADS-box TFs	Root development	Wang H. et al., 2016
miR528	MATE transporter family, Cu binding protein(CBF)	Enhances Tolerance to Salinity Stress and Nitrogen Starvation, Arsenite Tolerance	Liu et al., 2015; Yuan et al., 2015
miR529	SPL family	Phase transition	Morea et al., 2016
miR818	Serine/threonine kinase	Flowering time regulation	Liu and Zhang, 2012; Ding et al., 2013
miR827	ubiquitin E3 ligase	Suppress immune responses	Hewezi et al., 2016
miR2111	PHO2 and GmPT5	Responses to phosphate starvation	Xu et al., 2013
miR2118	MEL1 gene, TIR-NBS-LRR	Determinate fate acquisition of panicle meristems, drought stress responses	Wu et al., 2015; Ta et al., 2016

Other metals such as mercury, lead, and chromium have also been shown to affect miRNA expression. Mercury treatment differentially regulated miRNAs in *M. truncatula* where miR156, miR172, miR164, miR169, miR398 were downregulated whereas miR167 and miR172 were upregulated (Zhou et al., 2012). Cotton seedlings treated with Pb showed downregulation of miR156, miR398, miR399 and upregulation of miR162, miR167, miR169, miR395, miR396, and miR397 (He et al., 2014). Chromium showed upregulation of miR156, miR167, miR169, miR171, and downregulation of miR166 (Bukhari et al., 2015) (Figure 3). Among the heavy metal responsive miRNAs, miR156, miR159, miR166, and miR398 were shown to be differentially regulated. The currently available data about miRNA targets suggests that most of the miRNAs such as miR169, miR390, miR394, mir395, miR397, miR399, and miR528 are directly involved in the heavy metal stress tolerance by regulating the transcripts of ROS scavenging enzymes, laccases, or metal transporters. Apart from their direct involvement in heavy metal stress, some of the miRNAs play important role in plant growth and development. For example, miR156 regulate the important transitions in shoot development while miR159 is known to inhibit growth and promote programmed cell death by regulating R2R3 MYB transcription factors (Alonso-Peral et al., 2010; Xu et al., 2016) (Table 2). miR166 regulate diverse aspects like formation of apical and lateral meristem, leaf polarity vascular development, and floral development while miR398 is a major plant stress regulator (Jung and Park, 2007). Differential regulation of these important miRNAs by heavy metals might severely affect plant development by altering various mechanisms (Table 2).

A connection between miRNA and MAPK signaling was deciphered by a study which showed regulation of miR398b/c by OXI upon Cd and Cu treatment (Smeets et al., 2013). OXI is a component of MAPK cascade working upstream to MPK6, regulating ROS production (Asai et al., 2008) (Figure 2). Apart from this, several transcription factors which are known to be downstream target of MAPKs have also been found to be target of miRNA. Transcription factors of Squamosa promoter binding like protein (SPL) family, known to be involved in flower development, were studied to be the targets of miR156/157 and are regulated by Cd, Hg, and Al (Zhao et al., 2014) (Table 2). Additionally, SPLs binds to Cu responsive elements in the promoter of miR398 gene (Cu-responsive gene), regulating the expression of miR398 in Cu stress (Yamasaki et al., 2009). These studies uncover the concept of how plants exhibit interaction among different components in responding against the environmental stress.

### Modulation of transcription factors during heavy metal stress

Heavy metal toxicity is the serious problem of the modern world. For combating heavy metal stress, plants have evolved numerous detoxification and mobilization mechanisms as described earlier. Beside these, activation of complex signaling network is another important factor in heavy metal stress tolerance. Genome wide expression analysis have also reported modulation in the expression of transcription factor families upon exposure to heavy metals (Yanhui et al., 2006; Ogawa et al., 2009; Shim et al., 2009; Farinati et al., 2010; Wang et al., 2010; Smeets et al., 2013). Several studies have reported that upon heavy metal exposure, MAPK signaling cascade activates the downstream transcription factor targets (Figure 2). The transcription factors such as MYB (MYeloBlastosis), WRKY (containing a conserved WRKYGQK domain and a zinc finger-like motif), ZAT (C_2_H_2_-type zinc finger transcription factor), bZIP (basic region leucine ZIPper), AP2 (Activator Protein 2), ERF (ethylene-responsive factor), and DREB (dehydration responsive element-binding protein), have been identified as potential downstream targets of MAPKs (Roelofs et al., 2008; Li et al., 2016).

Transcription factors are important regulators of gene expression affecting many developmental processes and defense responses in plants (Yanhui et al., 2006). Studies have showed that upon exposure to Cd, expression of most of the transcription factors belonging to MYB, AP2, DREB, WRKY, and NAC up-regulates at different time intervals in rice (Ogawa et al., 2009). The MYB family is one of the largest families of transcription factors having diverse functions in eukaryotes (Dubos et al., 2010). Previous report have shown that MYBs such as MYB4, MYB28, MYB43, MYB48, MYB72, and MYB124 were highly induced in Cd and Zn metal stresses in Arabidopsis (van de Mortel et al., 2008). They have also found that the *MYB72* loss of function mutant exhibits increased metal sensitivity in Arabidopsis than the related Zn/Cd-hyper accumulator *Thlaspi caerulescens*. In another study, it was reported that Cd inactivates MYB2 by induction of NO production which causes nitrosylation of cysteine residues in the MYB2 transcription factor in Arabidopsis (Serpa et al., 2007). Recent reports have also established role of OsMYB45 in Cd toxicity (Hu et al., 2017). They found that mutation in *OsMYB45* resulted in Cd hypersensitive phenotype with significant increase in H_2_O_2_ content in the leaves of mutant and decrease in CAT activity as compared to the wild-type. In recent times, Wang F. Z. et al. (2017) have established the role of rice MYB transcription factor OsARM1 (ARSENITE-RESPONSIVE MYB1) that regulates As-associated transporters genes. He found that OsARM1 binds to the conserved MYB binding sites in the promoters of *OsLsi1, OsLsi2*, and *OsLsi6*, which encode key As transporters and affects their expression. Several studies suggest MYB TFs to be the downstream targets of MAPKs. Most recently, Li et al. (2016) found that MPK4 induced by light, regulates photoprotective anthocyanin biosynthesis by regulating MYB75/PAP1 transcription factor (Li et al., 2016). An altered gene expression and activity of MYBs as well as MAPKs in Cd stress gives us a clue of MYB being potential substrates of MAPKs during heavy metal stress.

WRKY transcription factors specifically bind to W-box in the promoters of many genes that are responsive to many biotic and abiotic environmental stress factors. Opdenakker et al. (2012) have reported significantly higher expression of members of WRKY family upon Cu and Cd metal exposures. They found that the transcription factors WRKY22, WRKY25, and WRKY29 were overexpressed in response to short-term exposure of roots to Cu. In contrast, only the expression of WRKY25 and WRKY29 affected upon exposure to Cd over a period of 24 hr (Opdenakker et al., 2012). In Cd treated *T. caerulescens*, expression of WRKY53 was found to be highly induced (Wei et al., 2008). Previous reports also showed that the flagellin induced MAPK cascade MEKK1-MKK4/MKK5-MPK3/MPK6 activates WRKY22 and its close homolog WRKY29 (Asai et al., 2002). Also there are reports showing SA dependent activation of WRKY25 and WRKY33 by MKS1 that directly interacts with MPK4 and negatively regulates defense responses in plants (Andreasson et al., 2005). In accordance with this, a tobacco WRKY1 was found to be phosphorylated by the defense-activating MAP-kinase SIPK (Menke et al., 2005). Also, it was reported that MPK3 and MPK6 phosphorylates WRKY33 and induces phytoalexin biosynthesis in Arabidopsis (Mao et al., 2011). Previous studies revealed that MEKK1 directly interact with WRKY53 on the protein level and also bind to its promoter (Miao et al., 2007). Most recently, expression of WRKY25, a downstream target for MPK4 was found to be up-regulated following Cd exposure (Smeets et al., 2013). Overall, these reports suggest that WRKY transcription factors can work coordinately with MAPK cascade during heavy metal stress tolerance.

Plant bZIP transcription factors are another class that provides defense against various environmental stresses including heavy metal stress. Reports have suggested induced expression of bZIP transcription factors upon Cd exposure (Ramos et al., 2007). Previous studies showed that the bZIP transcription factor from *B. juncea*, BjCdR15, is a regulator of Cd uptake, transport and accumulation in shoots and confers cadmium tolerance in transgenic plants (Farinati et al., 2010). In Soybean, Cd treatment significantly up-regulates bZIP62 expression while the expression of bZIP44 and bZIP78 is down-regulated (Chmielowska-Bak et al., 2014). Likewise, bZIP1 from *Tamarix hispida* showed increased expression in Cd stress in tobacco (Wang et al., 2010). Recent study on a novel bZIP gene, *BnbZIP3* from ramie (*Boehmeria nivea*) plant has showed that it positively regulates heavy metal stress tolerance by improving root growth upon overexpression (Huang et al., 2016). Though the direct link for bZIP transcription factors with MAPKs was not discovered in context to heavy metal stress but a report suggests that Arabidopsis bZIP transcription factor VIP1 (VirE1-Interacting Protein 1) localization and VirE2/T-DNA complex nuclear import may require phosphorylation by MPK3 (Djamei et al., 2007).

Apart from the above mentioned transcription factors, Cd also modulate expression of AP2/ERF family members, namely, ERF1 and ERF5 in Arabidopsis (Herbette et al., 2006). Similarly, Cd induces ERFs in *A. thaliana* and *A. halleri* (Weber et al., 2006). It has been reported that dehydration-responsive element-binding protein (DREB) transcription factors which are members of ERF family of transcription factors gets up-regulated upon heavy metal treatment. Cd leads to elevated expression of DREB1A and DREB1B in rice (Ogawa et al., 2009) while inhibited expression of transcription factors belonging to DRRB family was found in *Solanum torvum* plants (Yamaguchi et al., 2010). Recent studies have demonstrated that MPK3 and MPK6 regulates ethylene signaling by regulating ERF104 and Ethylene-Insensitive 3 (EIN3) which enhances the expression of ERF104 (Yoo et al., 2008; Bethke et al., 2009). In a recent study, a zinc finger transcription factor (C_2_H_2_-type), ZAT12 expression modulated upon short-term exposure to Cu while no such influence was observed upon long-term Cd exposure (Opdenakker et al., 2012). Recently, Arabidopsis ZAT6 was found to be positive regulator of Cd tolerance through the glutathione-dependent pathway (Chen et al., 2016). They identified that ZAT6 positively regulates expression of PCs synthesis pathway genes such as *GSH1, GSH2, PCS1*, and *PCS2*. Studies through protein-protein interaction also showed ZAT10 as direct substrate for MPK3 and MPK6 (Nguyen et al., 2012), suggesting involvement of MAPKs in regulation of heavy metal stress via ERFs and ZAT transcription factors.

Altogether, heavy metal stress activates various signaling components including MAPK cascades. Though, the reports on involvement of MAPKs upstream to transcription factors are rather scarce under metal stress but the above data demonstrated that they indeed interacts with transcription factors and mediates heavy metal stress tolerance response in plants.

### MAPK signaling in metal sequestration and transport

Encounter of heavy metal by plant roots generates many responses. This starts with binding of metal to the root cell wall and exudates, followed by metal influx across the plasma membrane. The high degree of metal influx is taken care by efflux of metal ions into the apoplast and chelation in the cytoplasm by PCs, metallothionines, organic, and amino acids. These metal ligand complexes are transported to the tonoplast and sequestered into the vacuoles (Sharma and Dietz, 2006; Verbruggen et al., 2009; Mendoza-Cózatl et al., 2011) (Figure 1). There are several molecules involved in this whole process of metal uptake, transportation, chelation, and sequestration. These are metal transporters and chelators accomplishing their task and protecting plants in metal toxicity (Singh S. et al., 2015).

MAPKs are one of the important signaling modules transmitting various stress related signals and are also known to get activated by heavy metal stress as discussed previously. The best characterized MAPKs MPK3 and MPK6 are the ones that are known to get expressed and activated by wide range of metals. Cadmium and copper have shown profound effect on these MAPKs in number of species. Up-regulation of MPK3 and MPK6 in these heavy metals gives us a clue about their function in metal homeostasis by either regulating downstream metal transporters or chelators that function in response to Cd and Cu. However, studies on ultimate effect of their activation on regulation of metal transporters, other TFs and proteins are still elusive in plants. Even though, there are significant reports on regulation of metal transporters by MAPKs in animals which gives an idea about this crosstalk occurring even in plants.

A number of metal transporters involved in metal ion homeostasis have been identified from different plants. The major groups of metal transporters studied are ZIP, heavy metal transport ATPase (CPx- and P1B-ATPase), NRAMP, CDF, and ABC transporters (Park et al., 2012; Singh S. et al., 2015). Several studies report their role in metal translocation and uptake based on expression pattern in different heavy metals. ZIP members were the first to be reported in plants, having ability to transport divalent cations like Zn^2+^, Fe^2+^, Mn^2+^, and Cd (Eide et al., 1996). IRT1 gene from Arabidopsis belonging to ZIP family is major transporter of iron leading to high affinity Fe uptake. In iron limiting environment IRT1 is present only in roots and is studied to be induced within 24 h of iron deficient conditions. Plants overexpressing IRT1 accumulate high levels of Cd and Zn along with Fe (Connolly et al., 2002). Other ZIP members ZIP1 and ZIP2 were studied to be Zn and Mn transporters in roots contributing to remobilization of Mn/Zn from vacuole to cytoplasm in root stellar cells and further movement from root stele to xylem parenchyma. According to their role, the expression of both the genes is mainly localized to the root stele (Milner et al., 2013). An interesting study on iron deficiency induced ethylene production in Arabidopsis reports the role of MPK3 and MPK6 in iron transport. The expression of iron transport and chelator genes (IRT1, FRO2, and FIT) was down regulated in *mpk3* and *mpk6* mutants under Fe deficiency (Ye et al., 2015), which suggests a possibility of these iron responsive genes working downstream of MAPK cascade (Table 3). This also suggests a crosstalk of MAPK signaling and hormone pathways in metal translocation in plants. Whilst in plants, there is single report of MAPK involvement in regulating metal transporter; in animals this concept is well-explored. It is not only the MAPKs known for regulating metal transporters but metal transporters are also equally involved in activating MAPKs. A report on chicken cell line suggest a role of ZIP transporter ZIP9/SLC39A9 in elevating intracellular zinc level and thereby regulating the activation of Erk MAPK signaling (Taniguchi et al., 2013) (Table 3).

**Table 3:** Metal transporters regulating MAPK signaling and vice versa.

**Family**		**Metal transporter**	**Metal ions**	**MAPKs**	**Model organism**	**Reference**
**METAL TRANSPORTERS REGULATING MAPKs**						
1	ZIP family	ZIP9/SLC39A9	Zn	ERK MAPKs	Chicken	Taniguchi et al., 2013
2	NRAMP family	NRAMP1 (SLC11A1)	–	P38MAPK	Mammals	Moisan et al., 2006
3	CTR family	CTR1	Cu	MEK1-ERK1	Mammals	Tsai et al., 2012; Turski et al., 2012
4	CDF family	ZnT1	Zn, Co, Cd	Raf1-MEK-ERK	*C. elegans*	Jirakulaporn and Muslin, 2004
5	Metallothioneins		Zn	P38 MAPK	Mammals	Rice et al., 2016
**MAPKs REGULATING METAL TRANSPORTERS**						
1	ZIP family	IRT1	Fe	MPK3, MPK6	Arabidopsis	Ye et al., 2015
2	NRAMP family	NRAMP1	–	P38MAPK, p42/44 MAPK	Mammals	Zhang et al., 2000
3	ABC family	MRP1	–	ERK/MAPK pathway	Mammals	El Azreq et al., 2012
4		ABCA1, ABCG1		Ras-MAPK pathway	Mammals	Mulay et al., 2013
5	Zn transporter family	Zrc1	Zn	Pbs2-Hog1-RCK1/RCK2	Yeast	Bilsland et al., 2004
6	Phytochelatins		–	Unknown MAPK	*S. mansoni*	Rigouin et al., 2013

Other metal transporter family NRAMP functions in diverse organisms ranging from bacteria to humans. In plants there are two subfamilies of NRAMP genes and several of them upregulate in Fe, Mn, and Cd deficiency. NRAMP proteins are studied to be localized on intracellular membranes of plastid and vacuolar membrane (Thomine and Schroeder, 2004). Expression analysis of NRAMP in plants suggests that unlike ZIP family (expressed mainly in roots) these metal transporters are expressed both in root and shoot, thus participating in metal homeostasis in all plant tissues. However, functional characterization of plant NRAMP transporters remains limited. Couple of studies in animal suggest the regulation of NRAMP1 transporter by MAPKs. This ion transporter was known to work downstream of p38 and p42/44 MAPK pathway activated upon proinflammatory mediators and bacterial infection in mammalian cells (Zhang et al., 2000). Also, another study implied the role of NRAMP1 in modulation of MAPK pathway (Moisan et al., 2006) (Table 3).

ABC transporters comprises of largest family, classified into eight subfamilies playing roles in diverse cellular processes like nutrient uptake, osmotic homeostasis, hormone transport, pathogen resistance, fatty acid import, and metal tolerance (Park et al., 2012). Arabidopsis ABC transporter, AtPDR8 is identified as cadmium extrusion pump conferring resistance to heavy metal Cd and Pb (Kim et al., 2007). Owing to their metal transportation capacity ABC C-type transporters AtABCC1 and AtABCC2 have been identified as major phytochelatin-heavy metal(oid) complex transporters. In recent study in wheat, expression of 13 ABC transporter genes was analyzed in different metals, which suggested that these genes were differentially regulated by Cd, indicating their participation in Cd uptake, transport, and sequestration (Wang X. et al., 2017). Yet another transporter of ABC family named as MRP1 (multidrug associated protein) is known to be regulated in ERK/MAPK pathway dependent manner in leukemic T cells (El Azreq et al., 2012). ABC transporters are known for their role in vacuole sequestration in plants. Besides this Ras/MAPK pathway is also reported to regulate ABC metal transporters (ABCA1 and ABCG1) in human hepatic cells (Mulay et al., 2013) (Table 3). A study in yeast proves the fact of activation of metal transporter by MAPKs more firmly. A MAPK cascade consisting of Pbs2-Hog1-Rck1/Rck2 in yeast is studied to activate a transcription factor (Yap2) and Zn transporter (Zrc1) thereby providing oxidative stress resistance (Bilsland et al., 2004) (Table 3).

Another metal transporter involves CTR transporter having an important role in maintaining Cu homeostasis in various species. CTR transporters are either plasma membrane proteins transporting Cu from extracellular spaces to cytosol or lysosome membrane proteins transporting Cu from lysosome to cytosol. In mammals, a study on high affinity copper transporter CTR1 was reported to activate MAPK cascade, wherein the mutation of CTR1 and Cu chelators reduces the activation of Erk1 (MAPK) by MEK1 (MAPKK) (Table 3). This is due to the fact that Erk1 phosphorylation by MEK1 requires Cu binding which diminishes in *ctr1* mutant (Tsai et al., 2012; Turski et al., 2012). Likewise, another report suggest that CDF proteins which are famous for the transport of Zn^2+^, Co^2+^, Cd in plants, modulates the activity of Raf1-MEK-ERK pathway in *C. elegans*. Its homolog in mammals, ZnT1 binds directly to the Raf1 in its regulatory domain thus activating it (Jirakulaporn and Muslin, 2004) (Table 3). Besides this, there are wide array of metal transporters that mediate Ca transport and its sequestration into the vacuole. Cation/proton exchangers (CAX) and isoforms have broad specificity and are widely implicated in Ca transport and other heavy metals like Mn^2+^ and Cd. CAX have been identified in salt tolerance, cadmium transport and tolerance. In mammals NHE1, one of the CAX was found to regulate MAPKs wherein it inhibited ERK1/2 and stimulated JNK1/2 activity (Pedersen et al., 2007).

Apart from the transporters activating MAPKs or vice versa, there are also studies suggesting the activation of MAPKs by metal chelators. The important metal chelators are PCs and metallothioneins and these are small cystein rich peptides with metal binding capacity (Singh S. et al., 2015). Two reports suggest MAPK activation by metallothioneins mainly by the release of the metal ions chelated (Chung et al., 2008; Rice et al., 2016). Additionally, PCs and phytochelatin synthase are hypothesized to be acting downstream of MAPK pathway in plants and human parasite respectively (Rigouin et al., 2013) (Table 3).

## Conclusion

From this review it is implied that metal exerts tremendous effect on plant by modulating its functioning at various levels. Metal stress activates several signaling pathways, known to have important role in imparting resistance against environmental stresses. Of these, an important signaling pathway contributing majorly in managing stress response is MAPK signaling pathway. Activation of signaling pathways magnifies the activation and functioning of various downstream components like transcription factors and other cytosolic protein thereby altering the expression of genes. In this review, the impact of heavy metals on activation of MAPK, calcium and hormone signaling along with other regulators like transcription factors and miRNAs is certainly reported. Several studies performed throughout different scientific groups suggest important role of MAPK signaling in heavy metal stress. However, a detailed evaluation of complete MAPK cascade working in combating heavy metal stress is required. Further, this review compiles the results revealing interplay of MAPK signaling with calcium, auxin, and ethylene signaling in response to heavy metal stress. All these findings outline the regulatory function of MAPKs acting either upstream or downstream to other signaling molecules. In animals this regulatory network has been elaborately studied in metal stress than in plants. Hence, it will be highly advantageous to study the importance of this signaling crosstalk in heavy metal stress in plants. Additionally, this review also summarizes interplay between MAPK signaling and other regulators like miRNAs and transcription factors in conveying a response against metal stress. However, scarce reports on regulatory network of MAPKs with transcription factors suggest a need for more in depth experiments in response to heavy metal stress in plants. Though, there are ample numbers of reports on activation of different signaling components, studies on deciphering of a complete regulatory network in heavy metal stress in plants are still lacking. Furthermore, the studies on activation of MAPKs by metals and metal transporters and in turn their regulation by MAPKs in animals and yeast, suggests occurrence of this phenomenon even in plants. However, the fragmentary work performed keeps this area mysterious in plants. Exploring the regulators of these metal transporters will contribute significantly in unraveling the mechanisms of metal stress tolerance in plants.

## Author contributions

SJ, PB, DV, SN, ST, and DS: wrote the manuscript; SJ: conceptualize the overall structure, prepared the illustrations, and edited the manuscript; KS and PB: prepared figures; AS: conceptualized, edited and approved the final manuscript.

### Conflict of interest statement

The authors declare that the research was conducted in the absence of any commercial or financial relationships that could be construed as a potential conflict of interest.

## References

[B1] Achard P. Herr A. Baulcombe D. C. Harberd N. P. (2004). Modulation of floral development by a gibberellin-regulated microRNA. Development 131, 3357–3365. 10.1242/dev.01206 15226253

[B2] Agrawal G. K. Iwahashi H. Rakwal R. (2003). Rice MAPKs. Biochem. Biophys. Res. Commun. 302, 171–180. 10.1016/S0006-291X(03)00174-8 12604328

[B3] Ahmad A. Hadi F. Ali N. (2015). Effective phytoextraction of cadmium (Cd) with increasing concentration of total phenolics and free proline in *Cannabis sativa* (L) plant under various treatments of fertilizers, plant growth regulators and sodium salt. Int. J. Phytoremed. 17, 56–65. 10.1080/15226514.2013.828018 25174425

[B4] Al-Hakimi A. M. A. (2007). Modification of cadmium toxicity in pea seedlings by kinetin. Plant Soil Environ. 53, 129–135. 10.4236/ajps.2016.712153

[B5] Alonso-Peral M. M. Li J. Li Y. Allen R. S. Schnippenkoetter W. Ohms S.. (2010). The microRNA159-regulated GAMYB-like genes inhibit growth and promote programmed cell death in Arabidopsis. Plant Physiol. 154, 757–771. 10.1104/pp.110.160630 20699403 PMC2949021

[B6] Andreasson E. Jenkins T. Brodersen P. Thorgrimsen S. Petersen N. H. Zhu S.. (2005). The MAP kinase substrate MKS1 is a regulator of plant defense responses. EMBO J. 24, 2579–2589. 10.1038/sj.emboj.7600737 15990873 PMC1176463

[B7] Arazi T. Sunkar R. Kaplan B. Fromm H. (1999). A tobacco plasma membrane calmodulin-binding transporter confers Ni^2+^ tolerance and Pb^2+^ hypersensitivity in transgenic plants. Plant J. 20, 171–182. 10.1046/j.1365-313x.1999.00588.x 10571877

[B8] Asai S. Ohta K. Yoshioka H. (2008). MAPK signaling regulates nitric oxide and NADPH oxidase dependent oxidative bursts in *Nicotiana benthamiana*. Plant Cell 20, 1390–1406. 10.1105/tpc.107.055855 18515503 PMC2438462

[B9] Asai T. Tena G. Plotnikova J. Willmann M. R. Chiu W. L. Gomez-Gomez L.. (2002). MAP kinase signalling cascade in Arabidopsis innate immunity. Nature 415, 977–983. 10.1038/415977a 11875555

[B10] Aukerman M. J. Sakai H. (2003). Regulation of flowering time and floral organ identity by a microRNA and its *APETALA2*-like target genes. Plant Cell 15, 2730–2741. 10.1105/tpc.016238 14555699 PMC280575

[B11] Bethke G. Unthan T. Uhrig J. F. Pöschl Y. Gust A. A. Scheel D.. (2009). Flg22 regulates the release of an ethylene response factor substrate from MAP kinase 6 in *Arabidopsis thaliana* via ethylene signaling. Proc. Natl. Acad. Sci. U.S.A. 106, 8067–8072. 10.1073/pnas.0810206106 19416906 PMC2683104

[B12] Bilsland E. Molin C. Swaminathan S. Ramne A. Sunnerhagen P. (2004). Rck1 and Rck2 MAPKAP kinases and the HOG pathway are required for oxidative stress resistance. Mol. Microbiol. 53, 1743–1756. 10.1111/j.1365-2958.2004.04238.x 15341652

[B13] Bukhari S. A. Shang S. Zhang M. Zheng W. Zhang G. Wang T. Z.. (2015). Genome-wide identification of chromium stress-responsive micro RNAs and their target genes in tobacco (*Nicotiana tabacum*) roots. Environ. Toxicol. Chem. 34, 2573–2582. 10.1002/etc.3097 26053264

[B14] Cao S. Chen Z. Liu G. Jiang L. Yuan H. Ren G.. (2009). The Arabidopsis Ethylene-Insensitive 2 gene is required for lead resistance. Plant Physiol. Biochem. 47, 308–312. 10.1016/j.plaphy.2008.12.013 19153049

[B15] Carrió-Seguí A. Garcia-Molina A. Sanz A. Peñarrubia L. (2015). Defective copper transport in the copt5 mutant affects cadmium tolerance. Plant Cell Physiol. 56, 442–454. 10.1093/pcp/pcu180 25432970

[B16] Chakrabarty D. Trivedi P. K. Misra P. Tiwari M. Shri M. Shukla D.. (2009). Comparative transcriptome analysis of arsenate and arsenite stresses in rice seedlings. Chemosphere 74, 688–702. 10.1016/j.chemosphere.2008.09.082 18996570

[B17] Chao S. H. Suzuki Y. Zysk J. R. Cheung W. Y. (1984). Activation of calmodulin by various metal cations as a function of ionic radius. Mol. Pharmacol. 26, 75–82. 6087119

[B18] Chen C. T. Chen L. Lin C. C. Kao C. H. (2001). Regulation of proline accumulation in detached rice leaves exposed to excess copper. Plant Sci. 160, 283–290. 10.1016/S0168-9452(00)00393-9 11164600

[B19] Chen J. Yang L. Yan X. Liu Y. Wang R. Fan T.. (2016). Zinc-Finger transcription factor ZAT6 positively regulates cadmium tolerance through the glutathione-dependent pathway in Arabidopsis. Plant Physiol. 171, 707–719. 10.1104/pp.15.01882 26983992 PMC4854688

[B20] Chen Y. A. Chi W. C. Trinh N. N. Huang L. Y. Chen Y. C. Cheng K. T.. (2014). Transcriptome profiling and physiological studies reveal a major role for aromatic amino acids in mercury stress tolerance in rice seedlings. PLoS ONE 9:e95163. 10.1371/journal.pone.0095163 24840062 PMC4026224

[B21] Chen Z. H. Bao M. L. Sun Y. Z. Yang Y. J. Xu X. H. Wang J. H. (2011). Regulation of auxin response by miR393-targeted transport inhibitor response protein1 is involved in normal development in Arabidopsis. Plant Mol. Biol. 77, 619–629. 10.1007/s11103-011-9838-1 22042293

[B22] Chiou T. J. Aung K. Lin S. I. Wu C. C. Chiang S. F. Su C. L. (2006). Regulation of phosphate homeostasis by microRNA in Arabidopsis. Plant Cell 18, 412–421. 10.1105/tpc.105.038943 16387831 PMC1356548

[B23] Chmielowska-Bak J. Gzyl J. Rucinska-Sobkowiak R. Arasimowicz-Jelonek M. Deckert J. (2014). The new insights into cadmium sensing. Front. Plant Sci. 5:245. 10.3389/fpls.2014.00245 24917871 PMC4042028

[B24] Choong G. Liu Y. Templeton D. M. (2014). Interplay of calcium and cadmium in mediating cadmium toxicity. Chem. Biol. Interact. 211, 54–65. 10.1016/j.cbi.2014.01.007 24463198

[B25] Chung R. S. Hidalgo J. West A. K. (2008). New insight into the molecular pathways of metallothionein-mediated neuroprotection and regeneration. J. Neurochem. 104, 14–20. 10.1111/j.1471-4159.2007.05026.x 17986229

[B26] Connolly E. L. Fett J. P. Guerinot M. L. (2002). Expression of the IRT1 metal transporter is controlled by metals at the levels of transcript and protein accumulation. Plant Cell 14, 1347–1357. 10.1105/tpc.001263 12084831 PMC150784

[B27] Cuypers A. Smeets K. Vangronsveld J. (2009). Heavy metal stress in plants, in Plant Stress Biology: From Genomics to Systems Biology, ed Hirt H. (Weinheim: Wiley-VCH Verlag GmbH & Co. KGaA), 161–178.

[B28] Debernardi J. M. Rodriguez R. E. Mecchia M. A. Palatnik J. F. (2012). Functional specialization of the plant miR396 regulatory network through distinct microRNA–target interactions. PLoS Genet. 8:e1002419. 10.1371/journal.pgen.1002419 22242012 PMC3252272

[B29] de la Torre F. Gutiérrez-Beltrán E. Pareja-Jaime Y. Chakravarthy S. Martin G. B. del Pozo O. (2013). The tomato calcium sensor Cbl10 and its interacting protein kinase Cipk6 define a signaling pathway in plant immunity. Plant Cell 25, 2748–2764. 10.1105/tpc.113.113530 23903322 PMC3753395

[B30] De Smet S. Cuypers A. Vangronsveld J. Remans T. (2015). Gene networks involved in hormonal control of root development in *Arabidopsis thaliana*: a framework for studying its disturbance by metal stress. Int. J. Mol. Sci. 16, 19195–19224. 10.3390/ijms160819195 26287175 PMC4581294

[B31] Ding H. Zhang A. Wang J. Lu R. Zhang H. Zhang J.. (2009). Identity of an ABA-activated 46 kDa mitogen-activated protein kinase from *Zea mays* leaves: partial purification, identification and characterization. Planta 230, 239–251. 10.1007/s00425-009-0938-y 19424717

[B32] Ding Y. Chen Z. Zhu C. (2011). Microarray-based analysis of cadmium-responsive microRNAs in rice (*Oryza sativa*). J. Exp. Bot. 62, 3563–3573. 10.1093/jxb/err046 21362738 PMC3130178

[B33] Ding Y. Qu A. Gong S. Huang S. Lv B. Zhu C. (2013). Molecular identification and analysis of Cd-responsive microRNAs in rice. J. Agricult. Food Chem. 61, 11668–11675. 10.1021/jf401359q 23909695

[B34] Ding Y. Ye Y. Jiang Z. Wang Y. Zhu C. (2016). MicroRNA390 Is involved in cadmium tolerance and accumulation in rice. Front. Plant Sci. 7:235. 10.3389/fpls.2016.00235 26973678 PMC4772490

[B35] Djamei A. Pitzschke A. Nakagami H. Rajh I. Hirt H. (2007). Trojan horse strategy in Agrobacterium transformation: abusing MAPK defense signaling. Science 318, 453–456. 10.1126/science.1148110 17947581

[B36] Dodd A. N. Kudla J. Sanders D. (2010). The language of calcium signaling. Annu. Rev. Plant Biol. 61, 593–620. 10.1146/annurev-arplant-070109-104628 20192754

[B37] Dubos C. Stracke R. Grotewold E. Weisshaar B. Martin C. Lepiniec L. (2010). MYB transcription factors in Arabidopsis. Trends Plant Sci. 15, 573–581. 10.1016/j.tplants.2010.06.005 20674465

[B38] Eide D. Broderius M. Fett J. Guerinot M. L. (1996). A novel iron-regulated metal transporter from plants identified by functional expression in yeast. Proc. Natl. Acad. Sci. U.S.A. 93, 5624–5628. 10.1073/pnas.93.11.5624 8643627 PMC39298

[B39] El Azreq M. A. Naci D. Aoudjit F. (2012). Collagen/β1 integrin signaling up-regulates the ABCC1/MRP-1 transporter in an ERK/MAPK-dependent manner. Mol. Biol. Cell 23, 3473–3484. 10.1091/mbc.E12-02-0132 22787275 PMC3431945

[B40] Ellis P. D. Strang P. Potter J. D. (1984). Cadmium-substituted skeletal troponin C. Cadmium-113 NMR spectroscopy and metal binding investigations. J. Biol. Chem. 259, 10348–10356. 6469967

[B41] Fahlgren N. Montgomery T. A. Howell M. D. Allen E. Dvorak S. K. Alexander A. L.. (2006). Regulation of AUXIN RESPONSE FACTOR3 by TAS3 ta-siRNA affects developmental timing and patterning in Arabidopsis. Curr. Biol. 16, 939–944. 10.1016/j.cub.2006.03.065 16682356

[B42] Fang H. Jing T. Liu Z. Zhang L. Jin Z. Pei Y. (2014). Hydrogen sulfide interacts with calcium signaling to enhance the chromium tolerance in *Setaria italica*. Cell Calcium 56, 472–481. 10.1016/j.ceca.2014.10.004 25459298

[B43] Fang Y. Xie K. Xiong L. (2014). Conserved miR164-targeted NAC genes negatively regulate drought resistance in rice. J. Exp. Bot. 65, 2119–2135. 10.1093/jxb/eru072 24604734 PMC3991743

[B44] Farinati S. DalCorso G. Varotto S. Furini A. (2010). The *Brassica juncea* BjCdR15, an ortholog of Arabidopsis TGA3, is a regulator of cadmium uptake, transport and accumulation in shoots and confers cadmium tolerance in transgenic plants. New Phytol. 185, 964–978. 10.1111/j.1469-8137.2009.03132.x 20028476

[B45] Fu S. F. Chen P. Y. Nguyen Q. T. T. Huang L. Y. Zeng G. R. Huang T. L.. (2014). Transcriptome profiling of genes and pathways associated with arsenic toxicity and tolerance in Arabidopsis. BMC Plant Biol. 14:94. 10.1186/1471-2229-14-94 24734953 PMC4021232

[B46] Gemrotová M. Kulkarni M. G. Stirk W. A. Strnad M. Van Staden J. Spíchal L. (2013). Seedlings of medicinal plants treated with either a cytokinin antagonist (PI-55) or an inhibitor of cytokinin degradation (INCYDE) are protected against the negative effects of cadmium. Plant Growth Regul. 71, 137–145. 10.1007/s10725-013-9813-8

[B47] Gong X. Liu Y. Huang D. Zeng G. Liu S. Tang H.. (2016). Effects of exogenous calcium and spermidine on cadmium stress moderation and metal accumulation in *Boehmeria nivea* (L.) Gaudich. Environ. Sci. Pollut. Res. 23, 8699–8708. 10.1007/s11356-016-6122-6 26801927

[B48] González A. Cabrera Mde L. Henríquez M. J. Contreras R. A. Morales B. Moenne A. (2012). Cross talk among calcium, hydrogen peroxide, and nitric oxide and activation of gene expression involving calmodulins and calcium-dependent protein kinases in *Ulva compressa* exposed to copper excess. Plant Physiol. 158, 1451–1462. 10.1104/pp.111.191759 22234999 PMC3291273

[B49] Gupta M. Sharma P. Sarin N. B. Sinha A. K. (2009). Differential response of arsenic stress in two varieties of *Brassica juncea* L. Chemosphere 74, 1201–1208. 10.1016/j.chemosphere.2008.11.023 19101007

[B50] Hamel L. P. Nicole M. C. Sritubtim S. Morency M. J. Ellis M. Ehlting J.. (2006). Ancient signals: comparative genomics of plant MAPK and MAPKK gene families. Trends Plant Sci. 11, 192–198. 10.1016/j.tplants.2006.02.007 16537113

[B51] Hamilton D. W. Hills A. Blatt M. R. (2001). Extracellular Ba^2+^ and voltage interact to gate Ca^2+^ channels at the plasma membrane of stomatal guard cells. FEBS Lett. 491, 99–103. 10.1016/S0014-5793(01)02176-7 11226428

[B52] He Q. Zhu S. Zhang B. (2014). MicroRNA–target gene responses to lead-induced stress in cotton (*Gossypium hirsutum* L.). Funct. Integr. Genomics 14, 507–515. 10.1007/s10142-014-0378-z 24879091

[B53] He X. Zheng W. Cao F. Wu F. (2016). Identification and comparative analysis of the microRNA transcriptome in roots of two contrasting tobacco genotypes in response to cadmium stress. Sci. Rep. 6:32805 10.1038/srep32805 27667199 PMC5036098

[B54] Herbette S. Taconnat L. Hugouvieux V. Piette L. Magniette M. L. Cuine S.. (2006). Genome-wide transcriptome profiling of the early cadmium response of *Arabidopsis* roots and shoots. Biochimie 88, 1751–1765. 10.1016/j.biochi.2006.04.018 16797112

[B55] Hewezi T. Piya S. Qi M. Balasubramaniam M. Rice J. H. Baum T. J. (2016). Arabidopsis miR827 mediates post-transcriptional gene silencing of its ubiquitin E3 ligase target gene in the syncytium of the cyst nematode *Heterodera schachtii* to enhance susceptibility. Plant J. 88, 179–192. 10.1111/tpj.13238 27304416

[B56] Hu S. Yu Y. Chen Q. Mu G. Shen Z. Zheng L. (2017). OsMYB45 plays an important role in rice resistance to cadmium stress. Plant Sci. 264, 1–8. 10.1016/j.plantsci.2017.08.002 28969789

[B57] Hu Y. F. Zhou G. Na X. F. Yang L. Nan W. B. Liu X.. (2013). Cadmium interferes with maintenance of auxin homeostasis in Arabidopsis seedlings. J. Plant Physiol. 170, 965–975. 10.1016/j.jplph.2013.02.008 23683587

[B58] Huang C. Zhou J. Jie Y. Xing H. Zhong Y. She W. (2016). A ramie (*Boehmeria nivea*) bZIP transcription factor BnbZIP3 positively regulates drought, salinity and heavy metal tolerance. Mol. Breed. 36:120 10.1007/s11032-016-0470-2

[B59] Huang J. Zhang Y. Peng J. S. Zhong C. Yi H. Y. Ow D. W.. (2012). Fission yeast HMT1 lowers seed cadmium through phytochelatin-dependent vacuolar sequestration in Arabidopsis. Plant Physiol. 158, 1779–1788. 10.1104/pp.111.192872 22319073 PMC3320185

[B60] Huang T. L. Huang H. J. (2008). ROS and CDPK-like kinase-mediated activation of MAP kinase in rice roots exposed to lead. Chemosphere 71, 1377–1385. 10.1016/j.chemosphere.2007.11.031 18164745

[B61] Huang T. L. Huang L. Y. Fu S. F. Trinh N. N. Huang H. J. (2014). Genomic profiling of rice roots with short-and long-term chromium stress. Plant Mol. Biol. 86, 157–170. 10.1007/s11103-014-0219-4 25056418

[B62] Jalmi S. K. Sinha A. K. (2015). ROS mediated MAPK signaling in abiotic and biotic stress-striking similarities and differences. Front. Plant Sci. 6:769. 10.3389/fpls.2015.00769 26442079 PMC4585162

[B63] Jirakulaporn T. Muslin A. J. (2004). Cation diffusion facilitator proteins modulate Raf-1 activity. J. Biol. Chem. 279, 27807–27815. 10.1074/jbc.M401210200 15096503

[B64] Jonak C. Nakagami H. Hirt H. (2004). Heavy metal stress. activation of distinct mitogen-activated protein kinase pathways by copper and cadmium. Plant Physiol. 136, 3276–3283. 10.1104/pp.104.045724 15448198 PMC523386

[B65] Jones D. L. Kochian L. V. (1997). Aluminum interaction with plasma membrane lipids and enzyme metal binding sites and its potential role in Al cytotoxicity. FEBS Lett. 400, 51–57. 10.1016/S0014-5793(96)01319-1 9000512

[B66] Jones-Rhoades M. W. Bartel D. P. (2004). Computational identification of plant microRNAs and their targets, including a stress-induced miRNA. Mol. Cell 14, 787–799. 10.1016/j.molcel.2004.05.027 15200956

[B67] Jung J. H. Park C. M. (2007). MIR166/165 genes exhibit dynamic expression patterns in regulating shoot apical meristem and floral development in Arabidopsis. Planta 225, 1327–1338. 10.1007/s00425-006-0439-1 17109148

[B68] Kende H. (1993). Ethylene biosynthesis. Annu. Rev. Plant Biol. 44, 283–307. 10.1146/annurev.pp.44.060193.001435

[B69] Keunen E. Schellingen K. Vangronsveld J. Cuypers A. (2016). Ethylene and metal stress: small molecule, big impact. Front. Plant Sci. 7:23. 10.3389/fpls.2016.00023 26870052 PMC4735362

[B70] Kim D. Y. Bovet L. Maeshima M. Martinoia E. Lee Y. (2007). The ABC transporter AtPDR8 is a cadmium extrusion pump conferring heavy metal resistance. Plant J. 50, 207–218. 10.1111/j.1365-313X.2007.03044.x 17355438

[B71] Kim S. H. Kim S. H. Yoo S. J. Min K. H. Nam S. H. Cho B. H.. (2013). Putrescine regulating by stress-responsive MAPK cascade contributes to bacterial pathogen defense in Arabidopsis. Biochem. Biophys. Res. Commun. 437, 502–508. 10.1016/j.bbrc.2013.06.080 23831467

[B72] Kong X. Zhang M. Xu X. Li X. Li C. Ding Z. (2014). System analysis of microRNAs in the development and aluminium stress responses of the maize root system. Plant Biotechnol. J. 12, 1108–1121. 10.1111/pbi.12218 24985700

[B73] Kovtun Y. Chiu W. L. Tena G. Sheen J. (2000). Functional analysis of oxidative stress-activated mitogen-activated protein kinase cascade in plants. Proc. Natl. Acad. Sci. U.S.A. 97, 2940–2945. 10.1073/pnas.97.6.2940 10717008 PMC16034

[B74] Krishnamurthy A. Rathinasabapathi B. (2013). Auxin and its transport play a role in plant tolerance to arsenite-induced oxidative stress in *Arabidopsis thaliana*. Plant Cell Environ. 36, 1838–1849. 10.1111/pce.12093 23489261

[B75] Kumar S. Dubey R. S. Tripathi R. D. Chakrabarty D. Trivedi P. K. (2015). Omics and biotechnology of arsenic stress and detoxification in plants: current updates and prospective. Environ. Int. 74, 221–230. 10.1016/j.envint.2014.10.019 25454239

[B76] Langer G. A. Nudd L. M. (1983). Effects of cations, phospholipases, and neuraminidase on calcium binding to “gas-dissected” membranes from cultured cardiac cells. Circ. Res. 53, 482–490. 10.1161/01.RES.53.4.482 6313248

[B77] Lequeux H. Hermans C. Lutts S. Verbruggen N. (2010). Response to copper excess in *Arabidopsis thaliana*: impact on the root system architecture, hormone distribution, lignin accumulation and mineral profile. Plant Physiol. Biochem. 48, 673–682. 10.1016/j.plaphy.2010.05.005 20542443

[B78] Li G. Meng X. Wang R. Mao G. Han L. Liu Y.. (2012). Dual-level regulation of ACC synthase activity by MPK3/MPK6 cascade and its downstream WRKY transcription factor during ethylene induction in Arabidopsis. PLoS Genet. 8:e1002767. 10.1371/journal.pgen.1002767 22761583 PMC3386168

[B79] Li S. Gao J. Yin K. Wang R. Wang C. Petersen M. (2016). MYB75 Phosphorylation by MPK4 is required for light-induced anthocyanin accumulation in Arabidopsis. Plant Cell 16:130 10.1105/tpc.16.00130 PMC5155340 27811015

[B80] Li W. X. Oono Y. Zhu J. He X. J. Wu J. M. Iida K.. (2008). The Arabidopsis NFYA5 transcription factor is regulated transcriptionally and posttranscriptionally to promote drought resistance. Plant Cell 20, 2238–2251. 10.1105/tpc.108.059444 18682547 PMC2553615

[B81] Li Z. Y. Xu Z. S. He G. Y. Yang G. X. Chen M. Li L. C.. (2012). Overexpression of soybean GmCBL1 enhances abiotic stress tolerance and promotes hypocotyl elongation in Arabidopsis. Biochem. Biophys. Res. Commun. 427, 731–736. 10.1016/j.bbrc.2012.09.128 23044418

[B82] Lima J. Arenhart R. A. Margis-Pinheiro M. Margis R. (2011). Aluminum triggers broad changes in microRNA expression in rice roots. Genet. Mol. Res. 10, 2817–2832. 10.4238/2011.November.10.4 22095606

[B83] Lin C. W. Chang H. B. Huang H. J. (2005). Zinc induces mitogen-activated protein kinase activation mediated by reactive oxygen species in rice roots. Plant Physiol. Biochem. 43, 963–968. 10.1016/j.plaphy.2005.10.001 16324848

[B84] Liu Q. Hu H. Zhu L. Li R. Feng Y. Zhang L.. (2015). Involvement of miR528 in the regulation of arsenite tolerance in rice (*Oryza sativa* L). J. Agric. Food Chem. 63, 8849–8861. 10.1021/acs.jafc.5b04191 26403656

[B85] Liu Q. Zhang H. (2012). Molecular identification and analysis of arsenite stress-responsive miRNAs in rice. J. Agric. Food Chem. 60, 6524–6536. 10.1021/jf300724t 22712679

[B86] Liu X. M. Kim K. E. Kim K. C. Nguyen X. C. Han H. J. Jung M. S.. (2010). Cadmium activates Arabidopsis MPK3 and MPK6 via accumulation of reactive oxygen species. Phytochemistry 71, 614–618. 10.1016/j.phytochem.2010.01.005 20116811

[B87] Liu Y. Zhang S. (2004). Phosphorylation of 1-aminocyclopropane-1-carboxylic acid synthase by MPK6, a stress-responsive mitogen-activated protein kinase, induces ethylene biosynthesis in Arabidopsis. Plant Cell 16, 3386–3399. 10.1105/tpc.104.026609 15539472 PMC535880

[B88] Luan S. Kudla J. Rodriguez-Concepcion M. Yalovsky S. Gruissem W. (2002). Calmodulins and calcineurin B–like proteins calcium sensors for specific signal response coupling in plants. Plant Cell 14, S389–S400. 10.1105/tpc.001115 12045290 PMC151268

[B89] Ma C. Burd S. Lers A. (2015). miR408 is involved in abiotic stress responses in Arabidopsis. Plant J. 84, 169–187. 10.1111/tpj.12999 26312768

[B90] Ma Z. Hu X. Cai W. Huang W. Zhou X. Luo Q.. (2014). Arabidopsis miR171-targeted scarecrow-like proteins bind to GT cis-elements and mediate gibberellin-regulated chlorophyll biosynthesis under light conditions. PLoS Genet. 10:e1004519. 10.1371/journal.pgen.1004519 25101599 PMC4125095

[B91] Maksymiec W. (2007). Signaling responses in plants to heavy metal stress. Acta Physiol. Plant. 29, 177–187. 10.1007/s11738-007-0036-3

[B92] Mao G. Meng X. Liu Y. Zheng Z. Chen Z. Zhang S. (2011). Phosphorylation of a WRKY transcription factor by two pathogen-responsive MAPKs drives phytoalexin biosynthesis in Arabidopsis. Plant Cell 23, 1639–1653. 10.1105/tpc.111.084996 21498677 PMC3101563

[B93] Marchant A. Kargul J. May S. T. Muller P. Delbarre A. Perrot-Rechenmann C.. (1999). AUX1 regulates root gravitropism in Arabidopsis by facilitating auxin uptake within root apical tissues. EMBO J. 18, 2066–2073. 10.1093/emboj/18.8.2066 10205161 PMC1171291

[B94] Matthewman C. A. Kawashima C. G. Húska D. Csorba T. Dalmay T. Kopriva S. (2012). miR395 is a general component of the sulfate assimilation regulatory network in Arabidopsis. FEBS Lett. 586, 3242–3248. 10.1016/j.febslet.2012.06.044 22771787

[B95] Mendoza-Cózatl D. G. Jobe T. O. Hauser F. Schroeder J. I. (2011). Long-distance transport, vacuolar sequestration, tolerance, and transcriptional responses induced by cadmium and arsenic. Curr. Opin. Plant Biol. 14, 554–562. 10.1016/j.pbi.2011.07.004 21820943 PMC3191310

[B96] Menke F. L. H. Kang H. G. Chen Z. Park J. M. Kumar D. Klessig D. F. (2005). Tobacco transcription factor WRKY1 is phosphorylated by the MAP kinase SIPK and mediates HR-like cell death in tobacco. Mol. Plant Microbe Interact. 18, 1027–1034. 10.1094/MPMI-18-1027 16255241

[B97] Miao Y. Laun T. M. Smykowski A. Zentgraf U. (2007). Arabidopsis MEKK1 can take a short cut: it can directly interact with senescence-related WRKY53 transcription factor on the protein level and can bind to its promoter. Plant Mol. Biol. 65, 63–76. 10.1007/s11103-007-9198-z 17587183

[B98] Mielniczki-Pereira A. A. Hahn A. B. B. Bonatto D. Riger C. J. Eleutherio E. C. A. Henriques J. A. P. (2011). New insights into the Ca^2+^-ATPases that contribute to cadmium tolerance in yeast. Toxicol. Lett. 207, 104–111. 10.1016/j.toxlet.2011.08.023 21911041

[B99] Milner M. J. Seamon J. Craft E. Kochian L. V. (2013). Transport properties of members of the ZIP family in plants and their role in Zn and Mn homeostasis. J. Exp. Bot. 64, 369–381. 10.1093/jxb/ers315 23264639 PMC3528025

[B100] Mockaitis K. Howell S. H. (2000). Auxin induces mitogenic activated protein kinase (MAPK) activation in roots of Arabidopsis seedlings. Plant J. 24, 785–796. 10.1046/j.1365-313x.2000.00921.x 11135112

[B101] Moisan J. Thuraisingam T. Henault J. De Sanctis J. Radzioch D. (2006). Role of SLC11A1 (formerly NRAMP1) in regulation of signal transduction induced by Toll-like receptor 7 ligands. FEMS Immunol. Med. Microbiol. 47, 138–147. 10.1111/j.1574-695X.2006.00077.x 16706797

[B102] Montero-Palmero M. B. Martín-Barranco A. Escobar C. Hernández L. E. (2014). Early transcriptional responses to mercury: a role for ethylene in mercury-induced stress. New Phytol. 201, 116–130. 10.1111/nph.12486 24033367

[B103] Morea E. G. O. da Silva E. M. e Silva G. F. F. Valente G. T. Rojas C. H. B. Vincentz M. (2016). Functional and evolutionary analyses of the miR156 and miR529 families in land plants. BMC Plant Biol. 16:40 10.1186/s12870-016-0716-5 26841873 PMC4739381

[B104] Mossor-Pietraszewska T. (2001). Effect of aluminium on plant growth and metabolism. Acta Biochim. Pol. 48, 673–686. 11833776

[B105] Mulay V. Wood P. Manetsch M. Darabi M. Cairns R. Hoque M.. (2013). Inhibition of mitogen-activated protein kinase Erk1/2 promotes protein degradation of ATP binding cassette transporters A1 and G1 in CHO and HuH7 cells. PLoS ONE 8:e62667. 10.1371/journal.pone.0062667 23634230 PMC3636258

[B106] Myouga F. Hosoda C. Umezawa T. Iizumi H. Kuromori T. Motohashi R.. (2008). A heterocomplex of iron superoxide dismutases defends chloroplast nucleoids against oxidative stress and is essential for chloroplast development in Arabidopsis. Plant Cell 20, 3148–3162. 10.1105/tpc.108.061341 18996978 PMC2613658

[B107] Nguyen X. C. Kim S. H. Lee K. Kim K. E. Liu X. M. Han H. J.. (2012). Identification of a C2H2-type zinc finger transcription factor (ZAT10) from Arabidopsis as a substrate of MAP kinase. Plant Cell Rep. 31, 737–745. 10.1007/s00299-011-1192-x 22134874

[B108] Nikovics K. Blein T. Peaucelle A. Ishida T. Morin H. Aida M.. (2006). The balance between the *MIR164A* and *CUC2* genes controls leaf margin serration in Arabidopsis. Plant Cell 18, 2929–2945. 10.1105/tpc.106.045617 17098808 PMC1693934

[B109] Noman A. Aqeel M. (2017). miRNA-based heavy metal homeostasis and plant growth. Environ. Sci. Pollut. Res. Int. 24, 10068–10082. 10.1007/s11356-017-8593-5 28229383

[B110] Ogawa I. Nakanishi H. Mori S. Nishizawa N. K. (2009). Time course analysis of gene regulation under cadmium stress in rice. Plant Soil 325:97 10.1007/s11104-009-0116-9

[B111] Opdenakker K. Remans T. Vangronsveld J. Cuypers A. (2012). Mitogen-activated protein (MAP) kinases in plant metal stress: regulation and responses in comparison to other biotic and abiotic stresses. Int. J. Mol. Sci. 13, 7828–7853. 10.3390/ijms13067828 22837729 PMC3397561

[B112] Osawa H. Matsumoto H. (2001). Possible involvement of protein phosphorylation in aluminum-responsive malate efflux from wheat root apex. Plant Physiol. 126, 411–420. 10.1104/pp.126.1.411 11351103 PMC102314

[B113] Pandey C. Raghuram B. Sinha A. K. Gupta M. (2015). miRNA plays a role in the antagonistic effect of selenium on arsenic stress in rice seedlings. Metallomics 7, 857–866. 10.1039/C5MT00013K 25772070

[B114] Park J. Song W. Y. Ko D. Eom Y. Hansen T. H. Schiller M.. (2012). The phytochelatin transporters AtABCC1 and AtABCC2 mediate tolerance to cadmium and mercury. Plant J. 69, 278–288. 10.1111/j.1365-313X.2011.04789.x 21919981

[B115] Pedersen S. F. Darborg B. V. Rasmussen M. Nylandsted J. Hoffmann E. K. (2007). The Na^+^/H^+^ exchanger, NHE1, differentially regulates mitogen-activated protein kinase subfamilies after osmotic shrinkage in Ehrlich Lettre Ascites cells. Cell. Physiol. Biochem. 20, 735–750. 10.1159/000110434 17982256

[B116] Peng J. S. Gong J. M. (2014). Vacuolar sequestration capacity and long-distance metal transport in plants. Front. Plant Sci. 5:19. 10.3389/fpls.2014.00019 24550927 PMC3912839

[B117] Perilli S. Moubayidin L. Sabatini S. (2010). The molecular basis of cytokinin function. Curr. Opin. Plant Biol. 13, 21–26. 10.1016/j.pbi.2009.09.018 19850510

[B118] Peto A. Lehotai N. Lozano-Juste J. León J. Tari I. Erdei L.. (2011). Involvement of nitric oxide and auxin in signal transduction of copper-induced morphological responses in Arabidopsis seedlings. Ann. Bot. 108, 449–457. 10.1093/aob/mcr176 21856638 PMC3158692

[B119] Piotrowska-Niczyporuk A. Bajguz A. Zambrzycka E. Godlewska-Zyłkiewicz B. (2012). Phytohormones as regulators of heavy metal biosorption and toxicity in green alga *Chlorella vulgaris* (*Chlorophyceae*). Plant Physiol. Biochem. 52, 52–65. 10.1016/j.plaphy.2011.11.009 22305067

[B120] Pitzschke A. Djamei A. Bitton F. Hirt H. (2009). A major role of the MEKK1–MKK1/2–MPK4 pathway in ROS signalling. Mol. Plant 2, 120–137. 10.1093/mp/ssn079 19529823 PMC2639734

[B121] Potters G. Pasternak T. P. Guisez Y. Palme K. J. Jansen M. A. (2007). Stress-induced morphogenic responses: growing out of trouble? Trends Plant Sci. 12, 98–105. 10.1016/j.tplants.2007.01.004 17287141

[B122] Raghuram B. Sheikh A. H. Sinha A. K. (2014). Regulation of MAP kinase signaling cascade by microRNAs in *Oryza sativa*. Plant signal. Behav. 9:e972130 10.4161/psb.29804 25482813 PMC4623436

[B123] Ramos J. Clemente M. R. Naya L. Loscos J. Perez-Rontome C. Sato S. (2007). Phytochelatin synthases of the model legume *Lotus japonicus*. a small multigene family with different responses to cadmium and alternatively spiced variants. Plant Physiol. 143, 110–118. 10.1104/pp.106.090894 PMC1820930 17208961

[B124] Rao K. P. Vani G. Kumar K. Wankhede D. P. Misra M. Gupta M.. (2011). Arsenic stress activates MAP kinase in rice roots and leaves. Arch. Biochem. Biophys. 506, 73–82. 10.1016/j.abb.2010.11.006 21081102

[B125] Rashotte A. M. Brady S. R. Reed R. C. Ante S. J. Muday G. K. (2000). Basipetal auxin transport is required for gravitropism in roots of Arabidopsis. Plant Physiol. 122, 481–490. 10.1104/pp.122.2.481 10677441 PMC58885

[B126] Rice J. M. Zweifach A. Lynes M. A. (2016). Metallothionein regulates intracellular zinc signaling during CD4^+^ T cell activation. BMC Immunol. 17:13. 10.1186/s12865-016-0151-2 27251638 PMC4890327

[B127] Rigouin C. Nylin E. Cogswell A. A. Schaumlöffel D. Dobritzsch D. Williams D. L. (2013). Towards an understanding of the function of the phytochelatin synthase of *Schistosoma mansoni*. PLoS Negl. Trop. Dis. 7:e2037. 10.1371/journal.pntd.0002037 23383357 PMC3561135

[B128] Rivetta A. Negrini N. Cocucci M. (1997). Involvement of Ca^2+^-calmodulin in Cd^2+^ toxicity during the early phases of radish (*Raphanus sativus* L.) seed germination. Plant Cell Environ. 20, 600–608. 10.1111/j.1365-3040.1997.00072.x

[B129] Rodriguez M. C. Petersen M. Mundy J. (2010). Mitogen-activated protein kinase signaling in plants. Annu. Rev. Plant Biol. 61, 621–649. 10.1146/annurev-arplant-042809-112252 20441529

[B130] Roelofs D. Aarts M. G. M. Schat H. Van Straalen N. M. (2008). Functional ecological genomics to demonstrate general and specific responses to abiotic stress. Funct. Ecol. 22, 8–18. 10.1111/j.1365-2435.2007.01312.x

[B131] Rucinska-Sobkowiak R. (2016). Water relations in plants subjected to heavy metal stresses. Acta Physiol. Plant. 38:257 10.1007/s11738-016-2277-5

[B132] Rudd J. J. Franklin-Tong V. E. (2001). Unravelling response-specificity in Ca^2+^ signalling pathways in plant cells. New Phytol. 151, 7–33. 10.1046/j.1469-8137.2001.00173.x 33873376

[B133] Salt D. E. Smith R. D. Raskin I. (1998). Phytoremediation. Annu. Rev. Plant Biol. 49, 643–668. 10.1146/annurev.arplant.49.1.643 15012249

[B134] Sanders D. Pelloux J. Brownlee C. Harper J. F. (2002). Calcium at the crossroads of signaling. Plant Cell 14, S401–S417. 10.1105/tpc.002899 12045291 PMC151269

[B135] Schellingen K. Van Der Straeten D. Remans T. Loix C. Vangronsveld J. Cuypers A. (2015). Ethylene biosynthesis is involved in the early oxidative challenge induced by moderate Cd exposure in *Arabidopsis thaliana*. Environ. Exp. Bot. 117, 1–11. 10.1016/j.envexpbot.2015.04.005 25743159

[B136] Schommer C. Palatnik J. F. Aggarwal P. Chételat A. Cubas P. Farmer E. E.. (2008). Control of jasmonate biosynthesis and senescence by miR319 targets. PLoS Biol. 6:e230. 10.1371/journal.pbio.0060230 18816164 PMC2553836

[B137] Schott E. J. Gardner R. C. (1997). Aluminum-sensitive mutants of *Saccharomyces cerevisiae*. Mol. Gen. Genet. 254, 63–72. 10.1007/s004380050391 9108291

[B138] Schwab R. Palatnik J. F. Riester M. Schommer C. Schmid M. Weigel D. (2005). Specific effects of microRNAs on the plant transcriptome. Dev. Cell 8, 517–527. 10.1016/j.devcel.2005.01.018 15809034

[B139] Serpa V. Vernal J. Lamattina L. Grotewold E. Cassia R. Terenzi H. (2007). Inhibition of AtMYB2 DNA-binding by nitric oxide involves cysteine S-nitrosylation. Biochem. Biophys. Res. Commun. 361, 1048–1053. 10.1016/j.bbrc.2007.07.133 17686455

[B140] Sethi V. Raghuram B. Sinha A. K. Chattopadhyay S. (2014). A mitogen-activated protein kinase cascade module, MKK3-MPK6 and MYC2, is involved in blue light-mediated seedling development in Arabidopsis. Plant Cell 26, 3343–3357. 10.1105/tpc.114.128702 25139007 PMC4371833

[B141] Sharma D. Tiwari M. Lakhwani D. Tripathi R. D. Trivedi P. K. (2015). Differential expression of microRNAs by arsenate and arsenite stress in natural accessions of rice. Metallomics 7, 174–187. 10.1039/C4MT00264D 25474357

[B142] Sharma S. S. Dietz K. J. (2006). The significance of amino acids and amino acid-derived molecules in plant responses and adaptation to heavy metal stress. J. Exp. Bot. 57, 711–726. 10.1093/jxb/erj073 16473893

[B143] Shen H. Hou N. Schlicht M. Wan Y. Mancuso S. Baluska F. (2008). Aluminium toxicity targets PIN2 in Arabidopsis root apices: effects on PIN2 endocytosis, vesicular recycling, and polar auxin transport. Chin. Sci. Bull. 53, 2480–2487. 10.1007/s11434-008-0332-3

[B144] Shim D. Hwang J. U. Lee J. Lee S. Choi Y. An G.. (2009). Orthologs of the class A4 heat shock transcription factor HsfA4a confer cadmium tolerance in wheat and rice. Plant Cell 21, 4031–4043. 10.1105/tpc.109.066902 20028842 PMC2814514

[B145] Siddiqui M. H. Al-Whaibi M. H. Sakran A. M. Basalah M. O. Ali H. M. (2012). Effect of calcium and potassium on antioxidant system of *Vicia faba* L. under cadmium stress. Int. J. Mol. Sci. 13, 6604–6619. 10.3390/ijms13066604 22837652 PMC3397484

[B146] Singh A. Roy S. Singh S. Das S. S. Gautam V. Yadav S.. (2017). Phytohormonal crosstalk modulates the expression of miR166/165s, target class III HD-ZIPs, and KANADI genes during root growth in *Arabidopsis thaliana*. Sci. Rep. 7:3408. 10.1038/s41598-017-03632-w 28611467 PMC5469759

[B147] Singh P. Mohanta T. K. Sinha A. K. (2015). Unraveling the intricate nexus of molecular mechanisms governing rice root development: OsMPK3/6 and auxin–cytokinin interplay. PLoS ONE 10:e0123620. 10.1371/journal.pone.0123620 25856151 PMC4391785

[B148] Singh S. Parihar P. Singh R. Singh V. P. Prasad S. M. (2015). Heavy metal tolerance in plants: role of transcriptomics, proteomics, metabolomics, and ionomics. Front. Plant Sci. 6:1143. 10.3389/fpls.2015.01143 26904030 PMC4744854

[B149] Sinha A. K. Jaggi M. Raghuram B. Tuteja N. (2011). Mitogen-activated protein kinase signaling in plants under abiotic stress. Plant Signal. Behav. 6, 196–203. 10.4161/psb.6.2.14701 21512321 PMC3121978

[B150] Smeets K. Opdenakker K. Remans T. Forzani C. Hirt H. Vangronsveld J.. (2013). The role of the kinase OXI1 in cadmium- and copper-induced molecular responses in *Arabidopsis thaliana*. Plant Cell Environ. 36, 1228–1238. 10.1111/pce.12056 23278806

[B151] Smith J. B. Dwyer S. D. Smith L. (1989). Cadmium evokes inositol polyphosphate formation and calcium mobilization. evidence for a cell surface receptor that cadmium stimulates and zinc antagonizes. J. Biol. Chem. 264, 7115–7118. 2540174

[B152] Song J. B. Gao S. Wang Y. Li B. W. Zhang Y. L. Yang Z. M. (2016). miR394 and its target gene LCR are involved in cold stress response in Arabidopsis. Plant Gene 5, 56–64. 10.1016/j.plgene.2015.12.001

[B153] Srivastava R. K. Pandey P. Rajpoot R. Rani A. Gautam A. Dubey R. S. (2015). Exogenous application of calcium and silica alleviates cadmium toxicity by suppressing oxidative damage in rice seedlings. Protoplasma 252, 959–975. 10.1007/s00709-014-0731-z 25413289

[B154] Srivastava S. Srivastava A. K. Suprasanna P. D'Souza S. F. (2013). Identification and profiling of arsenic stress-induced microRNAs in *Brassica juncea*. J. Exp. Bot. 64, 303–315. 10.1093/jxb/ers333 23162117

[B155] Steinhorst L. Jörg K. (2003). Calcium-a central regulator of pollen germination and tube growth. Biochimica et Biophysica Acta 1833, 1573–1581. 10.1016/j.bbamcr.2012.10.009 23072967

[B156] Steinhorst L. Kudla J. (2014). Signaling in cells and organisms–calcium holds the line. Curr. Opin. Plant Biol. 22, 14–21. 10.1016/j.pbi.2014.08.003 25195171

[B157] Stohs S. J. Bagchi D. (1995). Oxidative mechanisms in the toxicity of metal ions. Free Radic. Biol. Med. 18, 321–336. 10.1016/0891-5849(94)00159-H 7744317

[B158] Sun P. Tian Q. Y. Chen J. Zhang W. H. (2010). Aluminium-induced inhibition of root elongation in Arabidopsis is mediated by ethylene and auxin. J. Exp. Bot. 61, 347–356. 10.1093/jxb/erp306 19858117 PMC2803203

[B159] Sunkar R. Kaplan B. Bouche N. Arazi T. Dolev D. Talke I. N.. (2000). Expression of a truncated tobacco NtCBP4 channel in transgenic plants and disruption of the homologous Arabidopsis CNGC1 gene confer Pb^2+^ tolerance. Plant J. 24, 533–542. 10.1046/j.1365-313x.2000.00901.x 11115134

[B160] Ta K. N. Sabot F. Adam H. Vigouroux Y. De Mita S. Ghesquière A.. (2016). miR2118-triggered phased siRNAs are differentially expressed during the panicle development of wild and domesticated African rice species. Rice 9:10. 10.1186/s12284-016-0082-9 26969003 PMC4788661

[B161] Takahashi F. Mizoguchi T. Yoshida R. Ichimura K. Shinozaki K. (2011). Calmodulin-dependent activation of MAP kinase for ROS homeostasis in Arabidopsis. Mol. Cell 41, 649–660. 10.1016/j.molcel.2011.02.029 21419340

[B162] Tang M. Mao D. Xu L. Li D. Song S. Chen C. (2014). Integrated analysis of miRNA and mRNA expression profiles in response to Cd exposure in rice seedlings. BMC Genomics 15:835. 10.1186/1471-2164-15-835 25273267 PMC4193161

[B163] Taniguchi M. Fukunaka A. Hagihara M. Watanabe K. Kamino S. Kambe T.. (2013). Essential role of the zinc transporter ZIP9/SLC39A9 in regulating the activations of Akt and Erk in B-cell receptor signaling pathway in DT40 cells. PLoS ONE 8:e58022. 10.1371/journal.pone.0058022 23505453 PMC3591455

[B163a] Tchounwou P. B. Yedjou C. G. Patlolla A. K. Sutton D. J. (2012). Heavy metal toxicity and the environment. Mol. Clin. Environ. Toxicol. 101, 133–164. 10.1007/978-3-7643-8340-4_6 22945569 PMC4144270

[B164] Tebar F. Lladó A. Enrich C. (2002). Role of calmodulin in the modulation of the MAPK signalling pathway and the transactivation of epidermal growth factor receptor mediated by PKC. FEBS Lett. 517, 206–210. 10.1016/S0014-5793(02)02624-8 12062438

[B165] Thao N. P. Khan M. I. Thu N. B. Hoang X. L. Asgher M. Khan N. A.. (2015). Role of ethylene and its cross talk with other signaling molecules in plant responses to heavy metal stress. Plant Physiol. 169, 73–84. 10.1104/pp.15.00663 26246451 PMC4577409

[B166] Thomine S. Schroeder J. I. (2004). Plant metal transporters with homology to proteins of the NRAMP family, in The NRAMP family, eds Cellier M. Gros P. (Austin: Andes/Kluwer Series), 113–121.

[B167] Tian Q. Zhang X. Ramesh S. Gilliham M. Tyerman S. D. Zhang W. H. (2014). Ethylene negatively regulates aluminium-induced malate efflux from wheat roots and tobacco cells transformed with TaALMT1. J. Exp. Bot. 65, 2415–2426. 10.1093/jxb/eru123 24668874 PMC4036508

[B168] Tombuloglu G. Tombuloglu H. Sakcali M. S. Unver T. (2015). High-throughput transcriptome analysis of barley (*Hordeum vulgare*) exposed to excessive boron. Gene 557, 71–81. 10.1016/j.gene.2014.12.012 25498907

[B169] Trinh N. N. Huang T. L. Chi W. C. Fu S. F. Chen C. C. Huang H. J. (2014). Chromium stress response effect on signal transduction and expression of signaling genes in rice. Physiol. Plant. 150, 205–224. 10.1111/ppl.12088 24033343

[B170] Tripathi V. Parasuraman B. Laxmi A. Chattopadhyay D. (2009). CIPK6, a CBL-interacting protein kinase is required for development and salt tolerance in plants. Plant J. 58, 778–790. 10.1111/j.1365-313X.2009.03812.x 19187042

[B171] Tsai C. Y. Finley J. C. Ali S. S. Patel H. H. Howell S. B. (2012). Copper influx transporter 1 is required for FGF, PDGF and EGF-induced MAPK signaling. Biochem. Pharmacol. 84, 1007–1013. 10.1016/j.bcp.2012.07.014 22842628 PMC3464187

[B172] Tsai T. M. Huang H. J. (2006). Effects of iron excess on cell viability and mitogen-activated protein kinase activation in rice roots. Physiol. Plant. 127, 583–592. 10.1111/j.1399-3054.2006.00696.x

[B173] Turski M. L. Brady D. C. Kim H. J. Kim B. E. Nose Y. Counter C. M.. (2012). A novel role for copper in Ras/mitogen-activated protein kinase signaling. Mol. Cell. Biol. 32, 1284–1295. 10.1128/MCB.05722-11 22290441 PMC3302449

[B174] van de Mortel J. E. Schat H. Moerland P. D. Ver Loren van Themaat E. Van Der Ent S. Blankestijn H.. (2008). Expression differences for genes involved in lignin, glutathione and sulphate metabolism in response to cadmium in *Arabidopsis thaliana* and the related Zn/Cd-hyperaccumulator *Thlaspi caerulescens*. Plant Cell Environ. 31, 301–324. 10.1111/j.1365-3040.2007.01764.x 18088336

[B175] Vanstraelen M. Benková E. (2012). Hormonal interactions in the regulation of plant development. Annu. Rev. Cell Dev. Biol. 28, 463–487. 10.1146/annurev-cellbio-101011-155741 22856461

[B176] Vaucheret H. Vazquez F. Crété P. Bartel D. P. (2004). The action of ARGONAUTE1 in the miRNA pathway and its regulation by the miRNA pathway are crucial for plant development. Genes Dev. 18, 1187–1197. 10.1101/gad.1201404 15131082 PMC415643

[B177] Verbruggen N. Hermans C. Schat H. (2009). Mechanisms to cope with arsenic or cadmium excess in plants. Curr. Opin. Plant Biol. 12, 364–372. 10.1016/j.pbi.2009.05.001 19501016

[B178] Vitti A. Nuzzaci M. Scopa A. Tataranni G. Remans T. Vangronsveld J. (2013). Auxin and cytokinin metabolism and root morphological modifications in *Arabidopsis thaliana* seedlings infected with *Cucumber mosaic virus* (CMV) or exposed to cadmium. Int. J. Mol. Sci. 14, 6889–6902. 10.3390/ijms14046889 23531542 PMC3645669

[B179] Wang F. Z. Chen M. X. Yu L. J. Xie L. J. Yuan L. B. Qi H. (2017). OsARM1, an R2R3 MYB transcription factor, is involved in regulation of the response to arsenic stress in rice. Front. Plant Sci. 30:1868 10.3389/fpls.2017.01868 PMC5670359 29163593

[B180] Wang H. Jiao X. Kong X. Humaira S. Wu Y. Chen X.. (2016). A signaling cascade from miR444 to RDR1 in rice antiviral RNA silencing pathway. Plant Physiol. 170, 2365–2377. 10.1104/pp.15.01283 26858364 PMC4825140

[B181] Wang J. W. Wang L. J. Mao Y. B. Cai W. J. Xue H. W. Chen X. Y. (2005). Control of root cap formation by microRNA-targeted auxin response factors in Arabidopsis. Plant Cell 17, 2204–2216. 10.1105/tpc.105.033076 16006581 PMC1182483

[B182] Wang R. Wang J. Zhao L. Yang S. Song Y. (2015). Impact of heavy metal stresses on the growth and auxin homeostasis of Arabidopsis seedlings. Biometals 28, 123–132. 10.1007/s10534-014-9808-6 25416404

[B183] Wang S. Ren X. Huang B. Wang G. Zhou P. An Y. (2016). Aluminium-induced reduction of plant growth in alfalfa (*Medicago sativa*) is mediated by interrupting auxin transport and accumulation in roots. Sci. Rep. 6:30079. 10.1038/srep30079 27435109 PMC4951802

[B184] Wang X. Wang C. Sheng H. Wang Y. Zeng J. Kang H. (2017). Transcriptome-wide identification and expression analyses of ABC transporters in dwarf polish wheat under metal stresses. Biol. Plant. 61, 293–304. 10.1007/s10535-016-0697-0

[B185] Wang Y. Gao C. Liang Y. Wang C. Yang C. Liu G. (2010). A novel bZIP gene from *Tamarix hispida* mediates physiological responses to salt stress in tobacco plants. J. Plant Physiol. 167, 222–230. 10.1016/j.jplph.2009.09.008 19853962

[B187] Wang Y. Xu L. Chen Y. Shen H. Gong Y. Limera C.. (2013). Transcriptome profiling of radish (*Raphanus sativus* L.) root and identification of genes involved in response to lead (Pb) stress with next generation sequencing. PLoS ONE 8:e66539. 10.1371/journal.pone.0066539 23840502 PMC3688795

[B188] Weber M. Trampczynska A. Clemens S. (2006). Comparative transcriptome analysis of toxic metal responses in *Arabidopsis thaliana* and the Cd^2+^-hypertolerant facultative metallophyte *Arabidopsis halleri*. Plant Cell Environ. 29, 950–963. 10.1111/j.1365-3040.2005.01479.x 17087478

[B189] Wei W. Zhang Y. Han L. Guan Z. Chai T. (2008). A novel WRKY transcriptional factor from *Thlaspi caerulescens* negatively regulates the osmotic stress tolerance of transgenic tobacco. Plant Cell Rep. 27, 795–803. 10.1007/s00299-007-0499-0 18183400

[B190] Wu B. F. Li W. F. Xu H. Y. Qi L. W. Han S. Y. (2015). Role of cin-miR2118 in drought stress responses in Caragana intermedia and tobacco. Gene 574, 34–40. 10.1016/j.gene.2015.07.072 26216304

[B191] Wu Z. Liang F. Hong B. Young J. C. Sussman M. R. Harper J. F. (2002). An endoplasmic reticulum-bound Ca^2+^/Mn^2+^ pump, ECA1, supports plant growth and confers tolerance to Mn^2+^ stress. Plant Physiol. 130, 128–137. 10.1104/pp.004440 12226493 PMC166546

[B192] Wurzinger B. Mair A. Pfister B. Teige M. (2011). Cross-talk of calcium-dependent protein kinase and MAP kinase signaling. Plant Signal. Behav. 6, 8–12. 10.4161/psb.6.1.14012 21248475 PMC3121996

[B193] Xie Y. Hu L. Du Z. Sun X. Amombo E. Fan J.. (2014). Effects of cadmium exposure on growth and metabolic profile of bermudagrass [*Cynodon dactylon* (L.) Pers.]. PLoS ONE 9:e115279. 10.1371/journal.pone.0115279 25545719 PMC4278907

[B194] Xie Z. Kasschau K. D. Carrington J. C. (2003). Negative feedback regulation of dicer-like1 in Arabidopsis by microRNA-guided mRNA degradation. Curr. Biol. 13, 784–789. 10.1016/S0960-9822(03)00281-1 12725739

[B195] Xing Y. Chen W. Jia W. Zhang J. (2015). Mitogen-activated protein kinase kinase 5 (MKK5)-mediated signalling cascade regulates expression of iron superoxide dismutase gene in Arabidopsis under salinity stress. J. Exp. Bot. 66, 5971–5981. 10.1093/jxb/erv305 26136265 PMC4566985

[B196] Xu F. Liu Q. Chen L. Kuang J. Walk T. Wang J.. (2013). Genome-wide identification of soybean microRNAs and their targets reveals their organ-specificity and responses to phosphate starvation. BMC Genomics 14:66. 10.1186/1471-2164-14-66 23368765 PMC3673897

[B197] Xu J. Li Y. Wang Y. Liu H. Lei L. Yang H.. (2008). Activation of MAPK kinase 9 induces ethylene and camalexin biosynthesis and enhances sensitivity to salt stress in Arabidopsis. J. Biol. Chem. 283, 26996–27006. 10.1074/jbc.M801392200 18693252

[B198] Xu M. Hu T. Zhao J. Park M. Y. Earley K. W. Wu G.. (2016). Developmental functions of miR156-regulated squamosa promoter binding protein-like (SPL) genes in *Arabidopsis thaliana*. PLoS Genet. 12:e1006263. 10.1371/journal.pgen.1006263 27541584 PMC4991793

[B199] Yadav S. K. (2010). Heavy metals toxicity in plants: an overview on the role of glutathione and phytochelatins in heavy metal stress tolerance of plants. South Afr. J. Bot. 76, 167–179. 10.1016/j.sajb.2009.10.007

[B200] Yamaguchi H. Fukuoka H. Arao T. Ohyama A. Nunome T. Miyatake K.. (2010). Gene expression analysis in cadmium-stressed roots of a low cadmium-accumulating solanaceous plant, *Solanum torvum*. J. Exp. Bot. 61, 423–437. 10.1093/jxb/erp313 19837731 PMC2803209

[B201] Yamasaki H. Hayashi M. Fukazawa M. Kobayashi Y. Shikanai T. (2009). SQUAMOSA promoter binding protein–like7 is a central regulator for copper homeostasis in Arabidopsis. Plant Cell 21, 347–361. 10.1105/tpc.108.060137 19122104 PMC2648088

[B202] Yang T. Poovaiah B. W. (2003). Calcium/calmodulin-mediated signal network in plants. Trends Plant Sci. 8, 505–512. 10.1016/j.tplants.2003.09.004 14557048

[B203] Yanhui C. Xiaoyuan Y. Kun H. Meihua L. Jigang L. Zhaofeng G.. (2006). The MYB transcription factor superfamily of Arabidopsis: expression analysis and phylogenetic comparison with the rice MYB family. Plant Mol. Biol. 60, 107–124. 10.1007/s11103-005-2910-y 16463103

[B204] Ye L. Li L. Wang L. Wang S. Li S. Du J.. (2015). MPK3/MPK6 are involved in iron deficiency-induced ethylene production in Arabidopsis. Front. Plant Sci. 6:953. 10.3389/fpls.2015.00953 26579185 PMC4630569

[B205] Yeh C. M. Chien P. S. Huang H. J. (2007). Distinct signalling pathways for induction of MAP kinase activities by cadmium and copper in rice roots. J. Exp. Bot. 58, 659–671. 10.1093/jxb/erl240 17259646

[B206] Yoo S. D. Cho Y. H. Tena G. Xiong Y. Sheen J. (2008). Dual control of nuclear EIN3 by bifurcate MAPK cascades in C2H4 signalling. Nature 451, 789–795. 10.1038/nature06543 18273012 PMC3488589

[B207] Yu Y. Jin C. Sun C. Wang J. Ye Y. Zhou W.. (2016). Inhibition of ethylene production by putrescine alleviates aluminium-induced root inhibition in wheat plants. Sci. Rep. 6:8888. 10.1038/srep18888 26744061 PMC4705537

[B208] Yuan H. M. Huang X. (2016). Inhibition of root meristem growth by cadmium involves nitric oxide-mediated repression of auxin accumulation and signalling in Arabidopsis. Plant Cell Environ. 39, 120–135. 10.1111/pce.12597 26138870

[B209] Yuan S. Li Z. Li D. Yuan N. Hu Q. Luo H. (2015). Constitutive expression of rice MicroRNA528 alters plant development and enhances tolerance to salinity stress and nitrogen starvation in creeping bentgrass. Plant Physiol. 169, 576–593. 10.1104/pp.15.00899 26224802 PMC4577425

[B210] Zhang G. Wu H. Ross C. R. Ernest J. (2000). Cloning of porcine. Infect. Immun. 68, 1086–1093. 10.1128/IAI.68.3.1086-1093.2000 10678911 PMC97252

[B211] Zhang X. Wang L. Zhou A. Zhou Q. Huang X. (2016). Alterations in cytosol free calcium in horseradish roots simultaneously exposed to lanthanum (III) and acid rain. Ecotoxicol. Environ. Saf. 126, 62–70. 10.1016/j.ecoenv.2015.12.014 26720810

[B212] Zhao D. Li T. Shen M. Wang J. Zhao Z. (2015). Diverse strategies conferring extreme cadmium (Cd) tolerance in the dark septate endophyte (DSE), *Exophiala pisciphila*: evidence from RNA-seq data. Microbiol. Res. 170 27–35. 10.1016/j.micres.2014.09.005 25294257

[B213] Zhao F. Y. Wang K. Zhang S. Y. Ren J. Liu T. Wang X. (2014). Crosstalk between ABA, auxin, MAPK signaling, and the cell cycle in cadmium-stressed rice seedlings. Acta physiol. Plant. 36, 1879–1892. 10.1007/s11738-014-1564-2

[B214] Zhou Z. S. Zeng H. Q. Liu Z. P. Yang Z. M. (2012). Genome-wide identification of *Medicago truncatula* microRNAs and their targets reveals their differential regulation by heavy metal. Plant Cell Environ. 35, 86–99. 10.1111/j.1365-3040.2011.02418.x 21895696

